# Genetically inspired organoids prevent joint degeneration and alleviate chondrocyte senescence via Col11a1–HIF1α‐mediated glycolysis–OXPHOS metabolism shift

**DOI:** 10.1002/ctm2.1574

**Published:** 2024-02-05

**Authors:** Ye Sun, Yongqing You, Qiang Wu, Rui Hu, Kerong Dai

**Affiliations:** ^1^ Department of Orthopaedics The First Affiliated Hospital of Nanjing Medical University Jiangsu China; ^2^ Department of Orthopaedic Surgery Shanghai Key Laboratory of Orthopaedic Implants Shanghai Ninth People's Hospital, Shanghai Jiao Tong University School of Medicine Shanghai China; ^3^ Department of Renal Diseases Affiliated Hospital of Nanjing University of Chinese Medicine Nanjing China

**Keywords:** cellular senescence, genetics, glycolysis, hip dysplasia, organoids, osteoarthritis, single cell‐sequencing

## Abstract

**Introduction:**

Developmental dysplasia of hip (DDH) is a hip joint disorder leading to subsequent osteoarthritis. Previous studies suggested collagen XI alpha 1 (COL11A1) as a potential gene in hip dysplasia and chondrocyte degeneration. However, no genetic association has reported COL11A1‐related cellular therapy as treatment of DDH and joint degeneration.

**Methods and Results:**

We report identified genetic association between COL11A1 locus and DDH with genome‐wide association study (GWAS). Further exome sequencing for familial DDH patients was conducted in different populations to identify potential pathogenic Col11A1 variants for familiar DDH. Further studies demonstrated involvement of COL11A1 expression was down‐regulated in femoral head cartilage of DDH patients and Col11a1‐KO mice with induced DDH. Col11a1‐KO mice demonstrated aggravated joint degeneration and severe OA phenotype. To explore the underlying mechanism of Col11a1 in cartilage and DDH development, we generated scRNA‐seq profiles for DDH and Col11a1‐KO cartilage, demonstrating disrupted chondrocyte homeostasis and cellular senescence caused by Col11a1–HIF1α‐mediated glycolysis–OXPHOS shift in chondrocytes. Genetically and biologically inspired, we further fabricated an intra‐articular injection therapy to preventing cartilage degeneration by generating a Col11a1‐over‐expressed (OE) SMSC mini‐organoids. Col11a1‐OE organoids demonstrated superior chondrogenesis and ameliorated cartilage degeneration in DDH mice via regulating cellular senescence by up‐regulated Col11a1/HIF1α‐mediated glycolysis in chondrocytes.

**Conclusion:**

We reported association between COL11A1 loci and DDH with GWAS and exome sequencing. Further studies demonstrated involvement of COL11A1 in DDH patients and Col11a1‐KO mice. ScRNA‐seq for DDH and Col11a1‐KO cartilage demonstrated disrupted chondrocyte homeostasis and cellular senescence caused by Col11a1–HIF1α‐mediated glycolysis–OXPHOS shift in chondrocytes. Genetically and biologically inspired, an intra‐articular injection therapy was fabricated to prevent cartilage degeneration with Col11a1‐OE SMSC organoids. Col11a1‐OE organoids ameliorated cartilage degeneration in DDH mice via regulating cellular senescence by up‐regulated Col11a1/HIF1α‐mediated glycolysis in chondrocytes.

## Graphical abstract

Association between COL11A1 loci and DDH was identified with GWAS and exome sequencing. ScRNA‐seq for DDH and Col11a1‐KO cartilage demonstrated disrupted chondrocyte homeostasis and cellular senescence caused by glycolysisOXPHOS metabolism shift in chondrocytes. Genetically inspired, an intra‐articular injection therapy was fabricated to prevent cartilage degeneration with Col11a1‐OE SMSC organoids. Col11a1‐OE organoids ameliorated cartilage degeneration and inhibited cellular senescence by up‐regulated Col11a1/HIF1 α‐mediated glycolysis in chondrocytes.

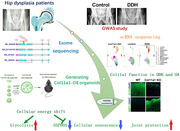

## 1 INTRODUCTION

Developmental dysplasia of hip (DDH) is a congenital osteoarticular disease, which causes dysplastic development of femoral head and acetabulum, resulting in hip semi‐dislocation at infancy and secondary osteoarthritis in adulthood.^1^, ^2^, ^3^ Early diagnosis and surgical correction of DDH is crucial to improve hip development and joint stability, preventing future joint degeneration of the patient and saving the medical expense of the afflicted family.^4^ Many risk factors have been reported for DDH.^5^ Environmental factors such as breech position, gestational age, oligohydramnios, infant position and nutrition factors may also contribute to DDH occurrence.^5^, ^6^, ^7^ Meanwhile, in DDH etiological research, many susceptibility genes have been revealed.^8^, ^9^, ^10^, ^11^ GDF5, TBX4, ASPN, CX3CR1 and other genes regulating osteochondral development and connective tissue formation have been reported for DDH in many populations.^8^, ^9^, ^12^, ^13^ Genetically directed therapy may provide a solution for DDH treatment and a preventive means for DDH‐related joint degeneration.

Collagen XI regulates the fibrillogenesis of collagen fibres as a fibril‐forming collagen.^14^ COL11A1 is essential for embryonic development and plays a crucial role in endochondral ossification. Hafez et al.^15^ revealed COL11A1 regulated bone microarchitecture at embryonic stage. Genetic COL11A1 mutations have been identified in many genetic diseases such as type 2 Stickler syndrome, Marshall syndrome and otospondylomegaepiphyseal dysplasia.^16^, ^17^ Meanwhile, COL11A1 mutant loci have been implicated in osteoarthritis, lumbar disc degeneration and other degenerative musculoskeletal diseases.^18^, ^19^ Mutations of COL11A1 has also been discovered in canine DDH, while no association has yet been reported in DDH patients.^20^

The treatment for DDH varies according to age and degrees of joint dysplasia. Common therapy for DDH including abduction orthosis, closed hip reduction, open hip reduction, femoral shortening osteotomy and pelvic osteotomy.^1^ Early treatment is essential to prevent joint degeneration in DDH patients. In clinical practice, arthroplasty may be indicated for severe secondary joint degeneration due to traumatic osteoarthritis in adulthood.^1^ Organoids have been applied in experimental treatment for tissue regeneration and self‐repair.^21^, ^22^ Meanwhile, preclinical application of organoids demonstrated good prospect as a biological treatment for osteoarthritis in mice.^23^, ^24^

In the current study, we reported genetic association between COL11A1 loci and DDH with genome‐wide association study (GWAS) and exome sequencing. Function of Col11a1 was further verified in DDH patients and DDH mice model with Col11a1 deficiency, which inspired us to explore the therapeutic effects of exploiting 3D‐cultured Col11a1‐over‐expressing (OE) organoids in preserving joint function and preventing secondary joint degeneration in DDH.

## 2 RESULTS

### 2.1 COL11A1 variants were associated with sporadic and familiar DDH in GWAS and exome sequencing

**FIGURE 1:**
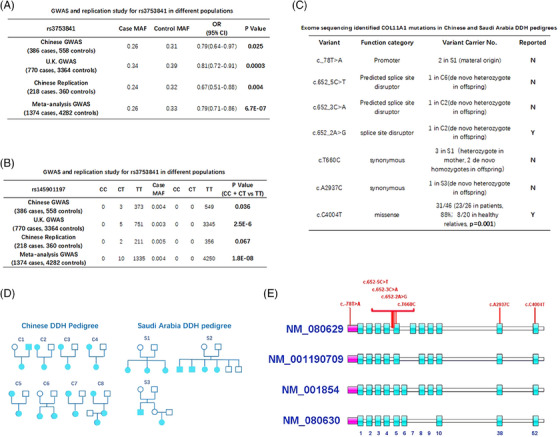
COL11A1 variants were associated with sporadic and familiar DDH in GWAS and exome sequencing. (A) GWAS and replication study for rs3753841 in different populations; (B) GWAS and replication study for rs145901197 in different populations; (C) exome sequencing identified COL11A1 mutations in (D) Chinese and Saudi Arabia DDH pedigrees; (E) whole exome sequencing found three novel de novo splicing site variants (c.652_5C>T, c.652_3C>A, c.652_2A>G), possibly leading to skip of exon 5 in splicing.

We examined DDH GWAS in the NJR and Chinese population that we previously performed.^25^, ^26^ Under the additive model, the COL11A1 locus rs3753841 was identified as a potential signal in the discovery stage for both GWAS (*p* = .025 in Chinese, *p* = .0003 in UK) (Figure 1A). Enlightened by early GWAS reports for this SNP in hip OA and hip dysplasia, a replication study was further conducted in a Chinese population comprising 218 cases and 360 controls and achieved a significant signal in replication (*p* = .003). Meta‐analysis for this variant in the discovering GWAS and replication was further conducted, achieving a *p* value of 6.7E−07 (Figure 1A). Another rare missense mutation, rs145901197 (Figure 1B) was identified under the dominant model in the UK GWAS dataset (MAF case vs. control: 0.0033 vs. 0, *p* = 2.5E−6) and Chinese GWAS dataset (MAF case vs. control: 0.0040 vs. 0, *p* = 2.5E−6). We also replicated rs145901197 in 218 cases and 360 controls (MAF case vs. control: 0.0047 vs. 0, *p* = .067; Figure 1B). Meta‐analysis of rs145901197 in 1374 cases and 4282 controls achieved genome‐wide significance (MAF case vs. control: 0.0037 vs. 0, *p* = 1.8E−8; Figure 1B). Crosscheck of the mutation frequency in gnomAD^27^ was made for rs145901197. The frequency of rs145901197 was six‐fold higher in the DDH sporadic patients than that in the gnomAD database (*C* = 0.0037, 10 out of 2680 in DDH; *C* = 0.00062, 152 out of 245 966 in gnomAD database). The sporadic DDH patients heterogenous for rs145901197 accounted for nearly 0.7% (10 carriers in 1345 DDH patients) of the DDH population. The exclusive rs145901197 mutation in DDH patients suggested the involvement of both the rare Col11A1 variant and the common variant in DDH. The rs145901197 is a missense variant predicted to be disease‐causing in the function prediction with both sorting intolerant from tolerant (SIFT^©^) algorithm and Mutation‐Taster^©^ program, leading to a change of Ile to Val in the amino acid sequence of Col11A1 transcript.

To further explore rare variants of Col11A1 in DDH heritability, exome sequencing for familial DDH patients was conducted in different populations (17 patients in eight Chinese families, nine patients in three Saudi Arabia families (Figures 1C–E). Exome findings of Col11A1 were extracted and validated by Sanger sequencing. The rare variant rs145901197 was not identified in DDH family members. Several potential pathogenic variants were identified in Col11A1 gene (Figure 1C). One mother‐originated rare promoter variant(c._78>A) was identified in S1 family. Five de novo variants (c.652_5C>T, c.652_3C>A, c.652_2A>G, c.T660C, c.A2937C) were identified in the DDH offspring generation amongst families. Three novel de novo splicing site variants (c.652_5C>T, c.652_3C>A, c.652_2A>G) were identified in two patients, which possibly lead to the skip of exon 5 in splicing. Interestingly. The splice site disruptor variant c.652_2A>G indeed can lead to splicing change and has been reported in a large family with hearing loss characterised with an inner ear cartilage anomaly,^28^ boosting confidence that the novel de novo Col11A1 splicing disruptors might be the causative variants for C2 and C6 DDH offspring. The missense variant rs3753841 was determined as deleterious in SIFT prediction. It was identified in 23 DDH familiar patients out of 26 patients in total, indicating the variant rs3753841 was present in all familiar DDH members except the S1 family. The frequency of rs3753841 variant carrier was significantly greater in familiar DDH members than in their relatives studied (23 out of 26 in patients, 88%; eight out of 20 in healthy relatives, 40%, *p* = .001) (Figures 1C–E).

### 2.2 COL11A1 expression was down‐regulated in joint cartilage tissues of DDH patients

**FIGURE 2:**
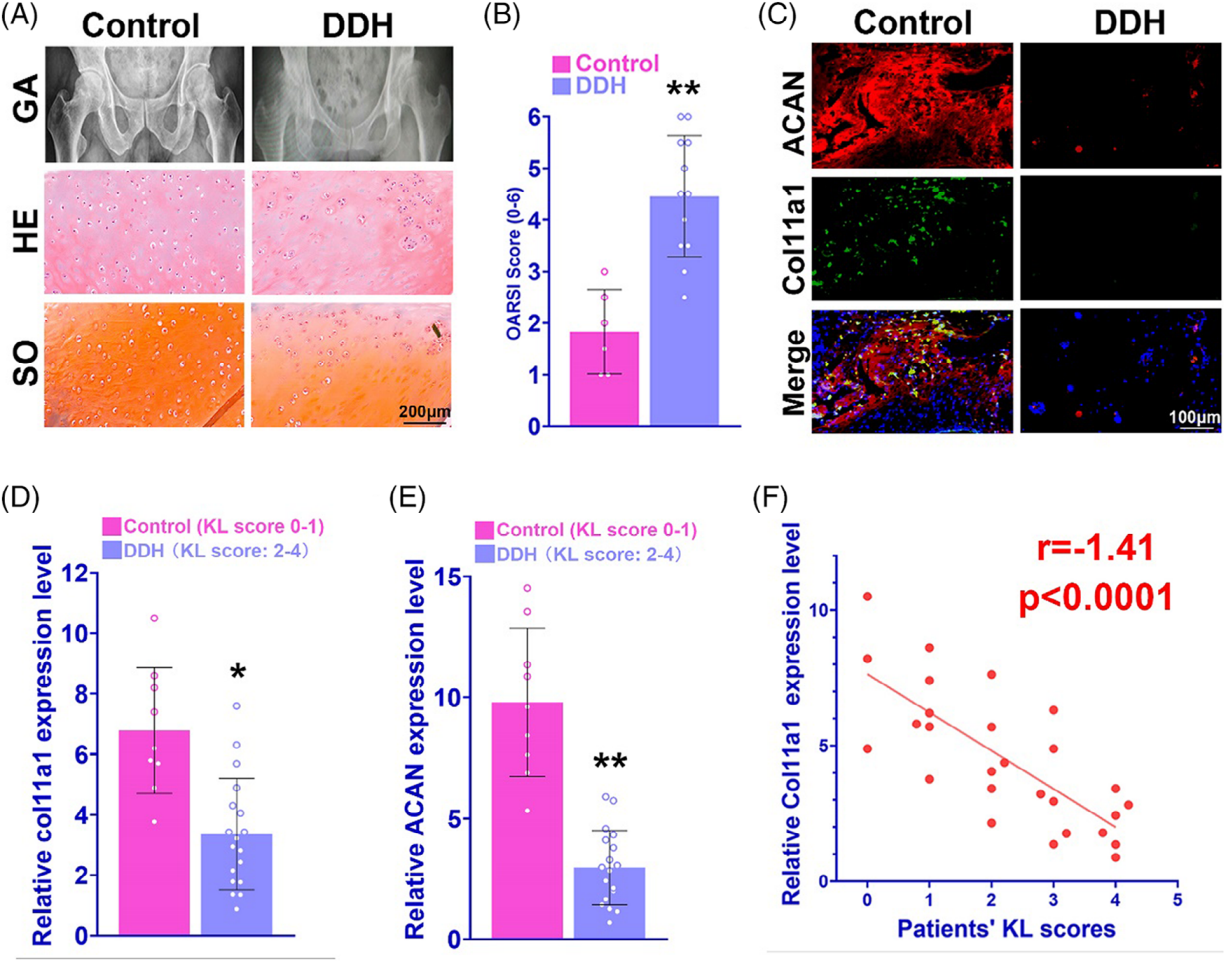
Difference of COL11A1 expression in the hip joint of DDH patients. (A) Pelvis X‐ray (AP view), H&E staining and Safranin‐O staining in sample of femoral head tissue of DDH patients and control. (B) OARSI score of DDH patients and control. (C) Immunofluorescence staining of ACAN and COL11A1 in sample of femoral head tissue of DDH patients and control. Reduction of COL11A1 and ACAN in sample of femoral head tissue of DDH patients (red, ACAN; green, COL11A1). (D) Relative COL11A1 expression level in femoral head tissue in DDH patients and control. (E) Relative ACAN expression level in femoral head tissue in DDH patients and control. (F) Relationship between DDH severity and COL11A1 expression with linear regression of patients’ Kellgren–Lawrence score and relative COL11A1 expression level (*n* = 27; *r* = −1.41, *p* < .0001). *p < .05, **p < .01 compared with the control group.

To explore the correlation between Col11a1 and DDH development, we collected 21 DDH cases (KL score = 1, three patients; KL socre = 2, six patients; KL score = 3, six patients; KL score = 4, six patients) and six control patients (KL score = 0, three patients; KL score = 1, three patients). The pelvis X‐ray confirmed DDH and severe hip joint degeneration (Figure 2A). To explore the histological differences between 12 severe DDH patients (KL score 3−4) and six control with trauma‐caused leg amputation, H&E and Safranin‐O staining were used to exam the femoral head cartilage samples, demonstrating less chondrocytes and lower cartilage matrix deposition in DDH patients (Figure 2A). The histopathological OARSI score of DDH group was significantly greater than the control (Figure 2B). Immunofluorescence staining also showed significantly lower ACAN and COL11A1 expression in DDH samples. (Figure 2C) Relative COL11A1 and ACAN gene expression levels were significantly lower in DDH group with 2−4 KL scores than control group (Figures 2D–E). Moreover, the COL11A1 expression level was negatively correlated with the joint degeneration grade, indicating COL11A1 expression could serve as a prognostic marker in DDH progression and hip joint degeneration (Figure 2F).

### 2.3 COL11A1 was down‐regulated in femoral head cartilage of surgical induced DDH mice model

**FIGURE 3:**
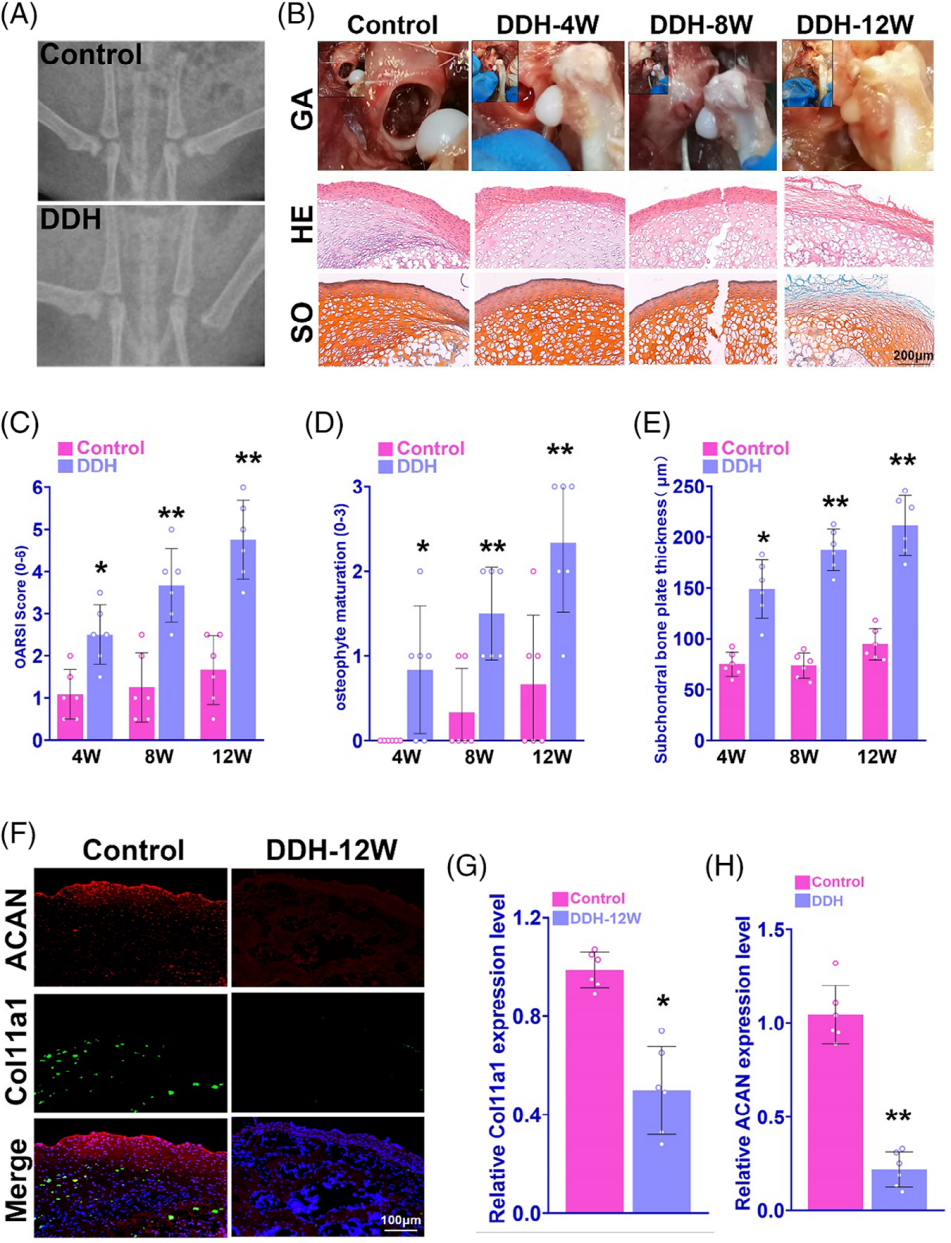
Dysregulation of COL11A1 expression in DDH mice model. (A) Pelvis X‐ray of DDH mice model and control. (B) Gross view and histological examination of DDH mice femoral head tissue over 12 weeks and control. H&E and Safranin‐O staining for tissue of mice femoral head. (C) OARSI histopathological score of DDH mice and control over 12 weeks (*n* = 6 for each). (D) Osteophyte maturation of DDH mice and control over 12 weeks. DDH group had greater extent of osteophyte maturation (*n* = 6 for each). (E) Subchondral bone plate thickness of DDH mice and control over 12 weeks. DDH group had greater subchondral bone plate thickness (*n* = 6 for each). (F) Immunofluorescence staining of ACAN and COL11A1 in DDH mice and control at the 12th week (red, ACAN; green, COL11A1). (G) Relative COL11A1 gene expression level in femoral head tissue in DDH mice and control. DDH group had higher relative COL11A1 expression level (*n* = 6 for each). (H) Relative ACAN expression level in femoral head tissue in DDH mice and control. DDH group had higher relative ACAN expression level (*n* = 6 for each). *p < .05, **p < .01 compared with the control group.

In DDH mice model, cartilage of femoral heads appeared to be morphologically abnormal and atrophic after surgical induction of DDH (Figure 3A). H&E and safranin‐O staining of femoral head slices demonstrated that DDH group showed tissue disintegratation, less chondrocytes and cartilage impairment compared with control group (Figure 3B). The OARSI score of DDH was significantly greater than the control studied after 12 weeks (Figure 3C). In DDH group, higher grade of osteophyte maturation and subchondral bone plate thickness (SBP) was observed compared with the control (Figures 3D and E). Immunofluorescence staining of chondrocyte marker indicated cartilage degeneration with less ACAN and COL11A1 expression in femoral head after 12 weeks in the DDH mice (Figure 3F). In qPCR analysis, relative COL11A1 and ACAN expression level were as well significantly lower in DDH group than control group (Figures 3G and H).

### 2.4 Col11a1‐KO mice demonstrated aggravated joint degeneration and severe OA phenotype

**FIGURE 4:**
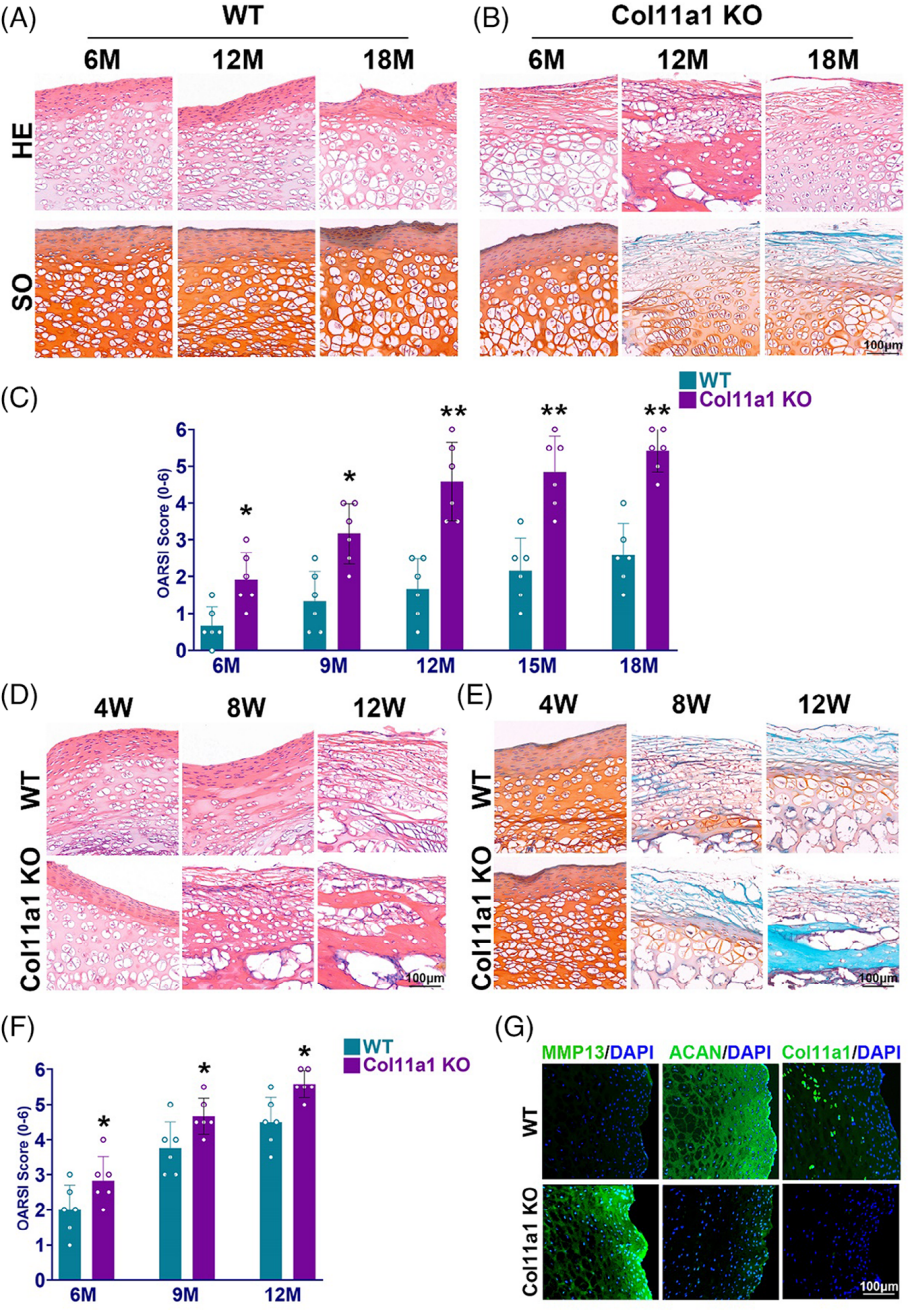
Severe joint degeneration was observed in Col11a1‐deficienct mice with induced DDH model. (A) H&E and Safranin‐O staining of femoral tissue in wide type mice at the 6th, 12th and 18th month. (B) H&E and Safranin‐O staining of femoral head tissue in COL11A1 knockout mice at the 6th, 12th and 18th month. (C) OARSI score of COL11A1 knockout and wide type mice over 18 months (*n* = 6 for each). (D) H&E staining of femoral head tissue in surgically induced DDH in COL11A1 knockout and wide type mice at the 4th, 8th and 12th week. (E) Safranin‐O staining of femoral tissue in surgically induced DDH in COL11A1 knockout and wide type mice at the over 12 weeks. (F) OARSI score in surgically induced DDH of COL11A1 knockout and wide type mice at the 6th, 9th and 12th month. (G) Immunofluorescence staining of MMP13 and ACAN expression in femoral head tissue in DDH mice and control at the 12‐month time point (green, MMP‐13, ACAN; blue, DAPI). *p < .05, **p < .01 compared with the control group.

Previous studies showed that the depletion of Col11a1 lead to a degenerative joint phenotype in mice. We hypothesise a potential degenerative effect might also be induced in DDH with Col11a1‐knockout mice. Col11a1‐KO mice was bred and observed at different time points for 18 months. In the 18‐month‐periods, Col11a1‐KO mice demonstrated greater histological hip joint degeneration and OA phenotype compared with wild‐type (WT) mice with H&E and safranin‐O staining (Figures 4A and B). To compare, Col11a1‐KO mice demonstrated significantly higher OASRI score than the WT group in the 18‐month‐period (Figure 4C). To verify the significance of Col11a1‐deficiency in DDH development, DDH was surgically induced and observed for 12 weeks in WT and Col11a1‐KO mice. In histological analysis, H&E and safranin‐O staining indicated that Col11a1‐KO mice showed increased joint degeneration with greater cartilage tissue damage, fewer chondrocytes and less chondral matrix deposition compared with WT DDH mice (Figures 4D and E). The phenomena were in consistent with the difference of OARSI score between WT and Col11a1‐KO, with the latter one being significantly higher (Figure 4F). Immunofluorescence staining showed disrupted cartilage anabolism and catabolism in Col11a1‐KO mice presenting with lower ACAN expression and significantly higher MMP‐13 expression than WT group (Figure 4G).

### 2.5 DDH and Col11a1‐KO cartilage demonstrated disrupted chondrocyte homeostasis and cellular similarity in single‐cell transcriptomic analysis

**FIGURE 5:**
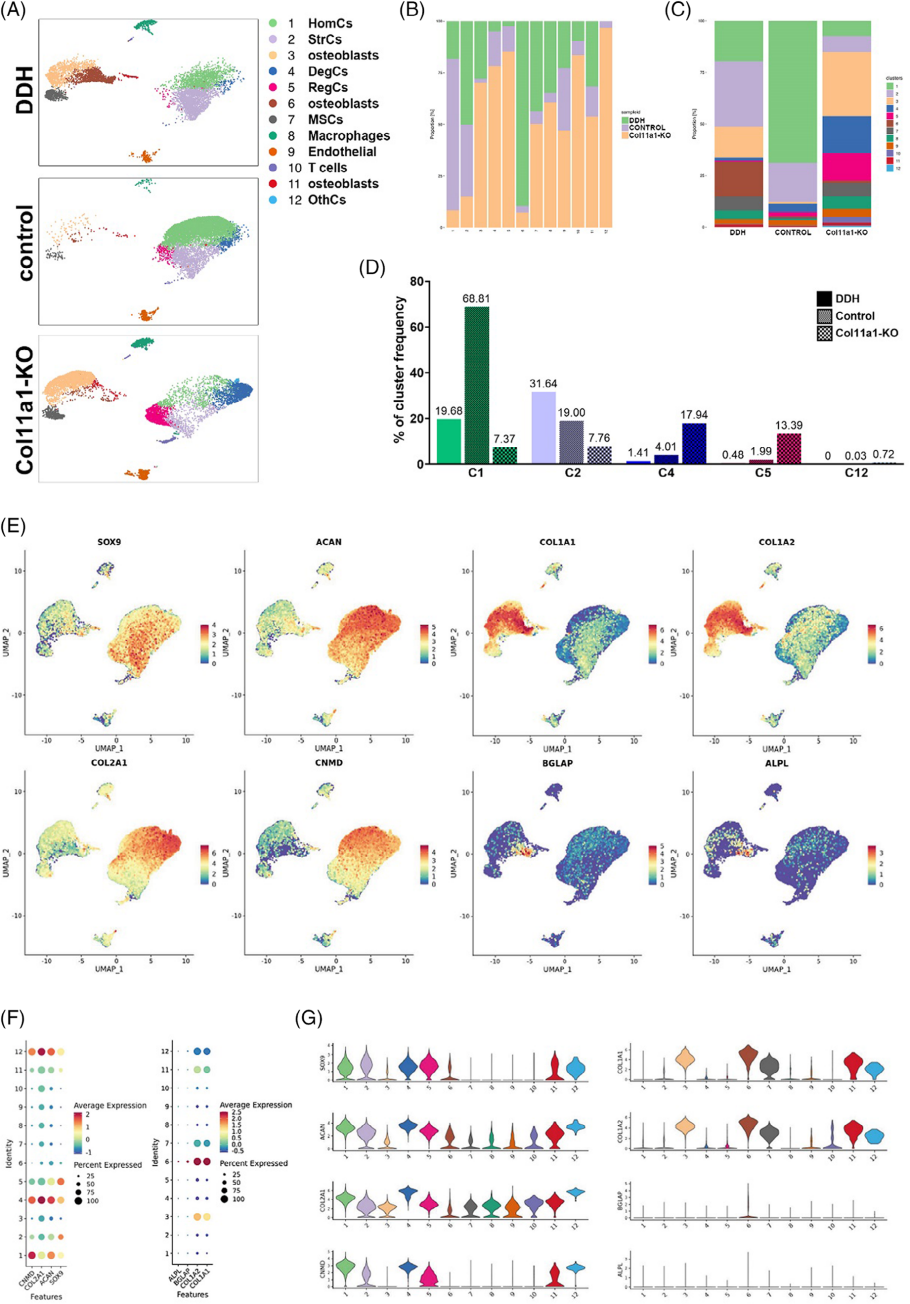
Single‐cell transcriptomic analysis of DDH, control and Col11a1‐KO cartilages. (A) The t‐distributed stochastic neighbour embedding (t‐SNE) plot of the five identified main chondrocyte clusters in cartilage samples. (B) Relative proportion of each cluster across three cartilage samples as indicated. (C) The frequency of each cluster in the DDH, control and Col11a1‐KO cartilages. (D) Frequency of each of five chondrocyte clusters in the DDH, control and Col11a1‐KO cartilages. (E) t‐SNE plots of the expression levels of chondrocyte and osteoblast marker genes. (F) Dot plot showing the mean expression of preferentially expressed chondrogenic and osteoblastic marker genes among the 12 clusters. (G) Violin plots demonstrating the normalised gene expression levels of representative marker genes among the clusters.

To explore the underlying mechanism of Col11a1 knockout in cartilage and DDH development, we generated scRNA‐seq profiles for hip joint cartilage of the control Group, the DDH group, and the Col11a1‐KO group (Figure 5A). According to the principle component analysis on gene expression patterns, 12 clusters of cells with different gene expression patterns were identified (Figures 5A–C). According to the t‐SNE analysis, five main unbiased chondrocyte clusters were identified based on their gene expression profiles and canonical markers (Figures 5A–C). Particularly, these cell clusters were: (C1) homeostatic chondrocytes (HomCs) preferentially expressing COL2A1, HAPLN1 and PRG4^29^, ^30^; (C2) stressed chondrocytes (StrCs) highly expressing MMP3, MMP13, CHI3L2 and ANGPTL4^31^; (C4) the degenerative chondrocytes (DegCs) specifically expressing RBP2, GSDMC, KCNA1 and PCP4^32^; (C5) the Regulatory chondrocytes (RegCs) with high expression of IGFBP1, SLC38A4, CHST13 and SLC2A12^33^ and (C12) the other chondrocytes (OthCs) with high expression of ARPP21, GRAMD2A, LBX1 and FAT3. We noticed that, in DDH and Col11a1‐KO cartilage, the frequency of HomCs was significantly declined (Figures 5B–D). DDH cartilage demonstrated higher proportions of StrCs, while the frequency of DegCs and RegCs were remarkably increased in Col11a1‐KO cartilage (Figure 5D), demonstrating imbalance of cartilage tissue homeostasis and severe joint degeneration in DDH and Col11a1‐KO cartilage. Moreover, the varied distribution of the four main chondrocyte clusters would suggest high heterogeneity of hip joint chondrocytes among the three samples (Figure 5D). In most cases, the well‐known chondrocyte markers were used in identifying specific types of cells, such as SOX9, ACAN, COL2A1 and CNMD for chondrocytes,^29^, ^33^ COL1A1, COL1A2, BGLAP and ALPL for osteoblasts^33^, ^34^ (Figures 5E–G). To further understand the difference between different chondrocyte clusters, the cluster marker gene data were analysed by Kyoto Encyclopedia of Genes and Genomes (KEGG) analysis (Figures S1A and B). It has been observed that the C1 cluster demonstrated significantly higher enrichment of genes related to TCA cycle, glycosaminoglycan biosynthesis, and metabolic pathways. In contrast, all of Cluster 2, 4 and 5 showed higher enrichment in gene concepts involved in oxidative phosphorylation (OXPHOS), mitophagy, cell cycle and ferroptosis (Figures S1B and D and S2). Gene set enrichment analysis also verified the alterations in the pathways related to TCA cycle, GAG biosynthesis, cell cycle, ferroptosis, metabolic pathways and OXPHOS in different chondrocyte clusters (Figures S1E–J), implying that the DDH development and Col11a1 deficiency would alter the chondrocyte cellular state by regulating these mentioned pathways above, leading to chondrocyte senescence, cartilage hypertrophy and joint degeneration. To get the overall characteristics of the cartilage, we further analysed specific gene expression patterns for the three groups (Figures S3A and B). KEGG analysis showed that DDH and Col11a1‐KO demonstrated significantly high similarity in enriched pathways, both leading to the differential expression of markers implicated in OXPHOS, mitophagy, hypoxia‐induced factor 1α (HIF‐1) signalling pathway, TGFβ signalling pathway, cellular senescence and apoptosis (Figures S3C and D). It further provided insights into the mechanism of Col11a1 deficiency in DDH development obtained in vitro and in vivo. Similarly, Gene Ontology (GO) analysis demonstrated enrichment in biological process related to growth plate cartilage chondrocyte morphogenesis, osteoblast differentiation, cartilage development, response to oxidative stress, response to hypoxia and apoptotic process (Figures S3E and F). Altogether the above results proved and provided evidence for Col11a1 deficiency in DDH development and joint degeneration in vitro and in vivo from the perspective of scRNA‐seq profiles, which demonstrated that the Col11a1 deficiency could impact chondrocyte homeostatic state by dysregulated TCA cycle with increased OXPHOS, alter the chondrocyte cluster frequency by decreased GAG biosynthesis, and impacting cell cycle with higher ferroptosis and cellular senescence to aggravate joint degeneration.

### 2.6 HIF1α‐mediated glycolysis–OXPHOS shift in DDH and Col11a1‐KO chondrocytes

**FIGURE 6:**
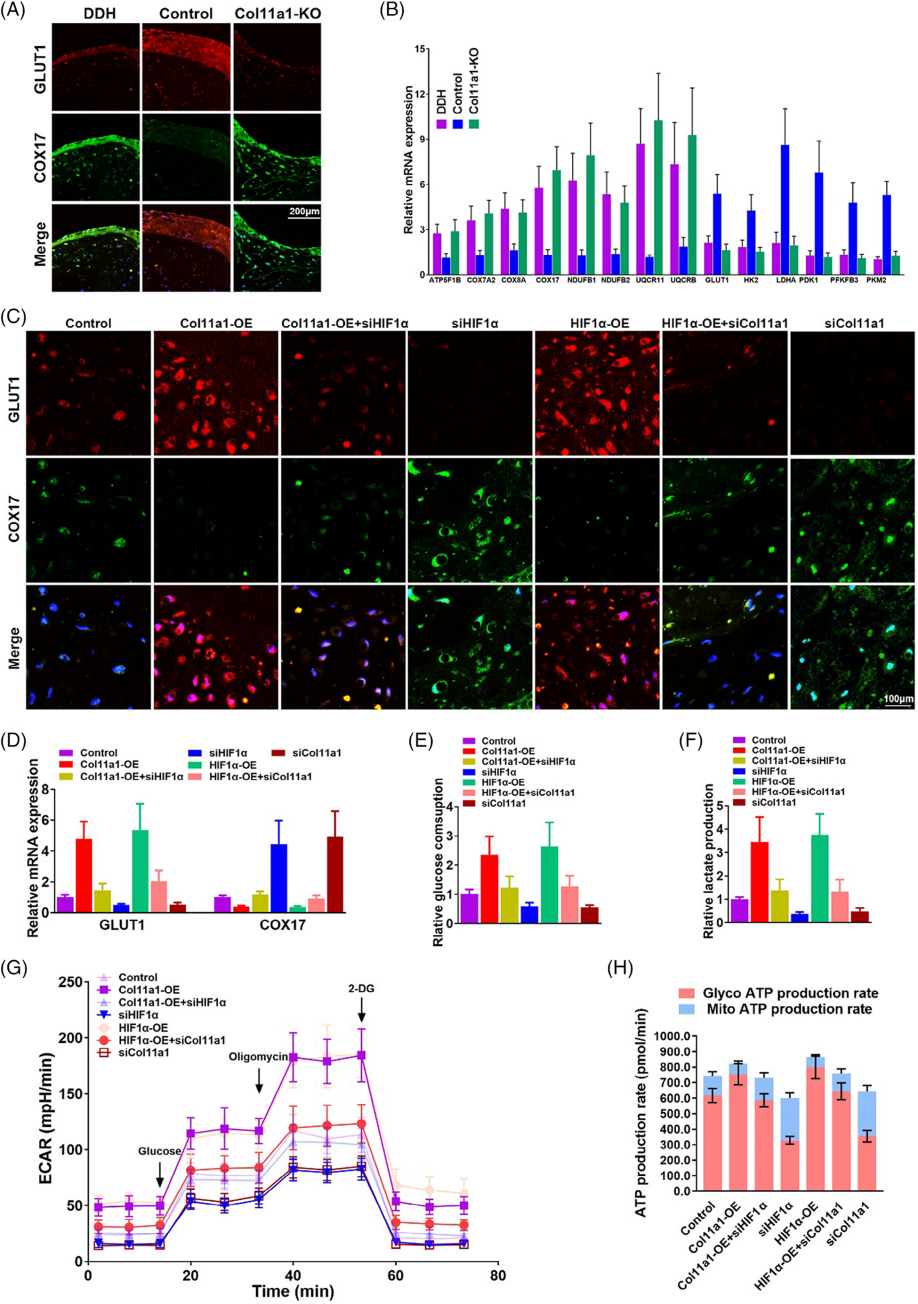
(A) Immunofluorescence staining of GLUT1 and COX17 expression in cartilage samples of DDH, Col11a1‐KO and control cartilage (green, COX17; red, GLUT1; blue, DAPI). (B) Relative glycolysis and OXPHOS marker gene expression levels quantified with qRT‐PCR in DDH, Col11a1‐KO and control cartilage. (C and D) Immunofluorescence staining and qRT‐PCR analysis of GLUT1 and COX17 in cultured chondrocytes transfected with HIF1α, Col11a1 small interfering RNA (siHIF1α and siCol11a1) construct, Lenti‐Col11a1 (Col11a1‐OE) and Lenti‐HIF1α (HIF1α‐OE) respectively in vitro. Col11a1‐OE and HIF1α‐OE chondrocytes were further transfected with siHIF1α and siCol11a1 respectively to verify the Col11a1/HIF1α axis. (E and F) Relative glucose consumption and lactate production by cultured chondrocytes with different treatments. (G) Extracellular acidification rate (ECAR) in glucose‐free medium was used to determine glycolytic metrics in treated chondrocytes, followed by sequential addition of glucose, oligomycin and 2‐deoxyglucose (2DG) (means ± SD, *n* = 6 for each). (H) Representative ATP production rate assay in chondrocytes wherein the contribution of glycolysis (pink bars) and mitochondrial respiration (blue bars) to ATP production after treatments for 24 h are as labelled (mean ± SD, *n* = 6).

Our results provided insights that the fine‐tuning between TCA cycle—OXPHOS in DDH and Col11a1‐KO chondrocytes might be an indispensable determinant of DDH and OA development. Glycolysis rather than OXPHOS in TCA cycle is the main metabolic energy source for chondrocytes in cartilage tissues due to the hypoxic microenvironment in vivo.^35^, ^36^ We hypothesised the shift of chondrocyte energy metabolism from glycolysis towards enhanced OXPHOS contributed to the chondrocyte cellular senescent state with Col11a1 deficiency. Interestingly, we found OXPHOS‐associated genes (e.g. COX17, ATP5F1B, COX7A2 …) were significantly up‐regulated and glycolysis‐associated genes (e.g. GLUT1, HK2, LDHA …) down‐regulated with Col11a1 deficiency (Figures 6A and B), which was consistent previous reports in cellular senescence and degenerative joint diseases.^37^, ^38^, ^39^ Our previous results showed that the HIF1α was an important upstream transcriptional regulator of chondrogenesis and joint cartilage formation.^24^ Meanwhile, our scRNA‐seq results demonstrated that genetic deletion of Col11a1 significantly suppressed HIF1 signalling pathway (Figure S3). Given the indispensable role of HIF1α in chondrocyte homeostasis and cellular glycolysis, we next investigated whether HIF1α mediated the glycolysis–OXPHOS reprogramming in Col11a1‐KO chondrocytes. First, we transfected chondrocytes with HIF1α and Col11a1 small interfering RNA (siHIF1α and siCol11a1) construct and demonstrated Col11a1 and HIF1α knockdown both led to decreased glycolysis with lower GLUT1 expression, lower cellular glucose consumption and lactate production (Figures 6C–F). Lenti‐Col11a1 (Col11a1‐OE) for Col11a1 overexpression significantly decreased COX17 expression and improved glycolysis with greater glucose consumption and lactate production (Figures 6C–F). Meanwhile, Lenti‐HIF1α (HIF1α‐OE) for HIF1α overexpression had the similar effects to Col11a1‐OE on chondrocytes. Moreover, HIF1α‐OE reversed the negative effects of siCol11a1 on cellular glycolysis (Figures 6C–F), suggesting Col11a1‐mediated chondrogenesis was mainly through the HIF1α/glycolysis pathway. We then used the Agilent Seahorse real‐time metabolic assays to investigate key parameters of the extracellular acidification rate (ECAR) during a glycolysis stress test of chondrocytes with siCol11a1, siHIF1α, Col11a1‐OE and HIF1α‐OE treatment respectively (Figures 6G and H). Indeed, the ECAR measured in the glycolysis stress test suggested significantly altered glycolytic patterns of chondrocytes treated with siCol11a1 or siHIF1α in comparison with the untreated cells (Figure 6G). In addition, both Col11a1‐OE and HIF1α‐OE showed increased glycolytic activities. Meanwhile, control, Col11a1‐OE+siHIF1α and HIF1α‐OE+si‐Col11a1 samples did not show significant differences in the glycolytic patterns, irrespective of the applied treatments (Figure 6G). Consequently, Col11a1/HIF1α axis activation led to higher ATP production, which was attributed to greater glycolytic ATP production and reduced mitochondrial ATP production by OXPHOS (Figure 6H). To verify the cellular state of chondrocytes with different treatments, Immunostaining of senescent markers HMGB1 and P16 was performed. As expected, Col11a1–HIF1α axis activation significantly prevented cellular senescence with greater HMGB1 expression and lower P16 expression (Figure S4). In conclusion, activating Col11a1/HIF1α axis appeared to be an anti‐senescent target for DDH treatment and OA prevention by regulating chondrocyte metabolism with glycolysis–OXPHOS reprogramming. Strategies boosting Col11a1 expression might potentiate cellular therapies for DDH and secondary OA.

### 2.7 Col11a1‐OE SMSC organoids demonstrated superior chondrogenic properties in vitro

Results above showed COL11A1 deficiency would lead to joint degeneration and chondrocyte senescence through HIF1α‐mediated glycolysis–OXPHOS reprogramming. We hypothesised that cellular therapies with boosted Col11a1 expression might be promising in the treatment of DDH and secondary OA.

**FIGURE 7:**
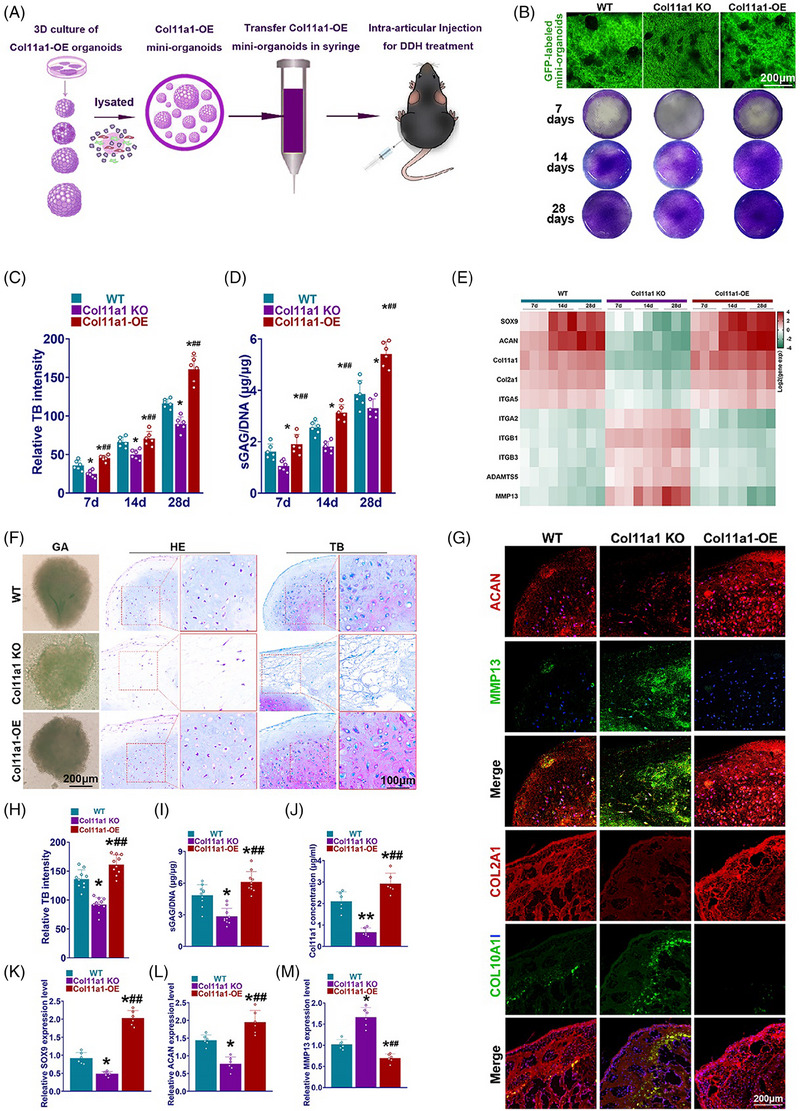
Schematic diagrams and characterisation for intra‐articular Col11a1‐OE organoids injection treatment for DDH. (A) Schematic illustration of the study design with 3D cultured Col11a1‐OE organoids for DDH treatment by intra‐articular injection in mice. (B) Toluidine blue staining (lower column) of GFP‐labelled organoids in different groups (upper column) over 28 days in vitro. (C) Relative toluidine blue intensity of COL11A1 KO, COL11A1 OE and WT organoids after 7, 14 and 28 days of in vitro culture (*n* = 6 for each). (D) GAG deposition of COL11A1 KO, COL11A1 OE and WT organoids after 7, 14 and 28 days of in vitro culture (*n* = 6 for each). (E) Expression of hyaline‐cartilage and fibro‐cartilage markers to validate the heterogeneity of phenotypes in the COL11A1 KO, COL11A1 OE and WT organoids after 7, 14 and 28 days of in vitro culture (*n* = 3 for each). *p < .05, **p < .01 compared with the WT group, #*p* < .05, ##*p* < .01 compared with the Col11a1‐KO group. (F) Gross view of organoids, H&E and toluidine blue staining of organoids in vitro culture. Wide type organoids were oval, COL11A1 KO organoids were irregular, COL11A1 organoids were spherical. Col11a1‐KO organoids generated less cartilage and collagen than WT organoids, Col11a1‐OE organoids generated more cartilage and collagen than WT organoids. (G) Immunofluorescence staining of ACAN,MMP‐13,COL2A1 and COL10A1 in organoids in vitro culture (red, ACAN and COL2A1; green, MMP‐13 and COL10A1; blue, DAPI). (H) Relative toluidine blue intensity of WT, COL11A1 KO and Col11a1‐OE organoids (*n* = 6 for each). (I) sGAG/DNA ratio of WT, COL11A1 KO and Col11a1‐OE organoids (*n* = 6 for each). (J–M) COL11A1 concentration, relative SOX9, ACAN and MMP‐13 expression level in organoids in vitro culture (*n* = 6 for each). *p < .05, **p < .01 compared with the WT group, #*p* < .05, ##*p* < .01 compared with the Col11a1‐KO group.

Our previous study showed chondrogenic organoids could promote cartilage repair and reverse OA development. To verify our hypothesis, we fabricated the Col11a1‐OE SMSC organoids and explored its potential application in DDH and OA treatment (Figures 7A and B). The Col11a1‐OE SMSCs were 3D‐cultured to generate organoids to implant in vivo for 28 days. Then the organoids were dissolved into mini‐ organoids as previous described.^24^ The Col11a1‐OE organoids were transferred in syringe and injected into the mice hip articular cavity for DDH treatment. We generated 3D‐cultured SMSC organoids for potential applications in the treatment of DDH and OA. Chondrogenesis was analysed for the organoids showing significantly greater expression of the classical chondrogenic markers (Figures 7C–E). Col11a1‐OE organoids demonstrated higher relative toluidine blue intensity and sGAG/DNA deposition (Figures 7C and D). Heatmap of gene expression profile showed that Col11a1‐OE organoids had higher expression of hyaline‐cartilage marker (Figure 7E). Col11a1‐KO organoids expressed significant higher fibrocartilage and degeneration/catabolic markers (Figure 7E). SMSCs auto‐assembled and form a stack to attain the spheroid shape in the 28‐day culture, suggesting the compaction of organoids with confined cartilage deposition (Figure 7F). Col11a1‐OE organoids were spherical while Col11a1‐KO organoids demonstrated irregular shape (Figure 7F). Slices of organoids also showed different component in different groups. H&E staining showed Col11a1‐OE organoids had highest cellular intensity compared with the control group and Col11a1‐KO organoids (Figure 7F). Toluidine blue staining showed significant difference in cartilage matrix deposition between Col11a1‐OE organoids and other groups (Figures 7F–H). Chondrogenic ability measured by sGAG/DNA deposition and COL11A1 concentration was significantly different among these groups (Figures 7I and J). Chondrogenic properties of different organoids were next analysed with qRT‐PCR for expression of chondrogenic and hypertrophic markers (SOX9, ACAN, MMP13) (Figures 7K–M). Compared with the other two groups, Col11a1‐OE group led to significantly better chondrogenesis evidenced with higher SOX9 and ACAN expression. Meanwhile, significant decrease in MMP13 expression was observed for Col11a1‐OE group, indicating that COL11A1‐OE could suppress chondrocyte hypertrophy and probably delay secondary joint degeneration (Figures 7K–M). Immunofluorescence staining further proved significant chondrogenic differences by greater ACAN and COL2A1 expression with much lower MMP13 and COL10A1 expression between Col11a1‐OE group and other groups of organoids (Figures 7G and K–M). All these phenomena demonstrated superior chondrogenic properties for Col11a1‐OE organoids, suggesting its potential in mitigating joint degeneration.

### 2.8 Col11a1‐OE organoids ameliorated cartilage degeneration in DDH mice

**FIGURE 8:**
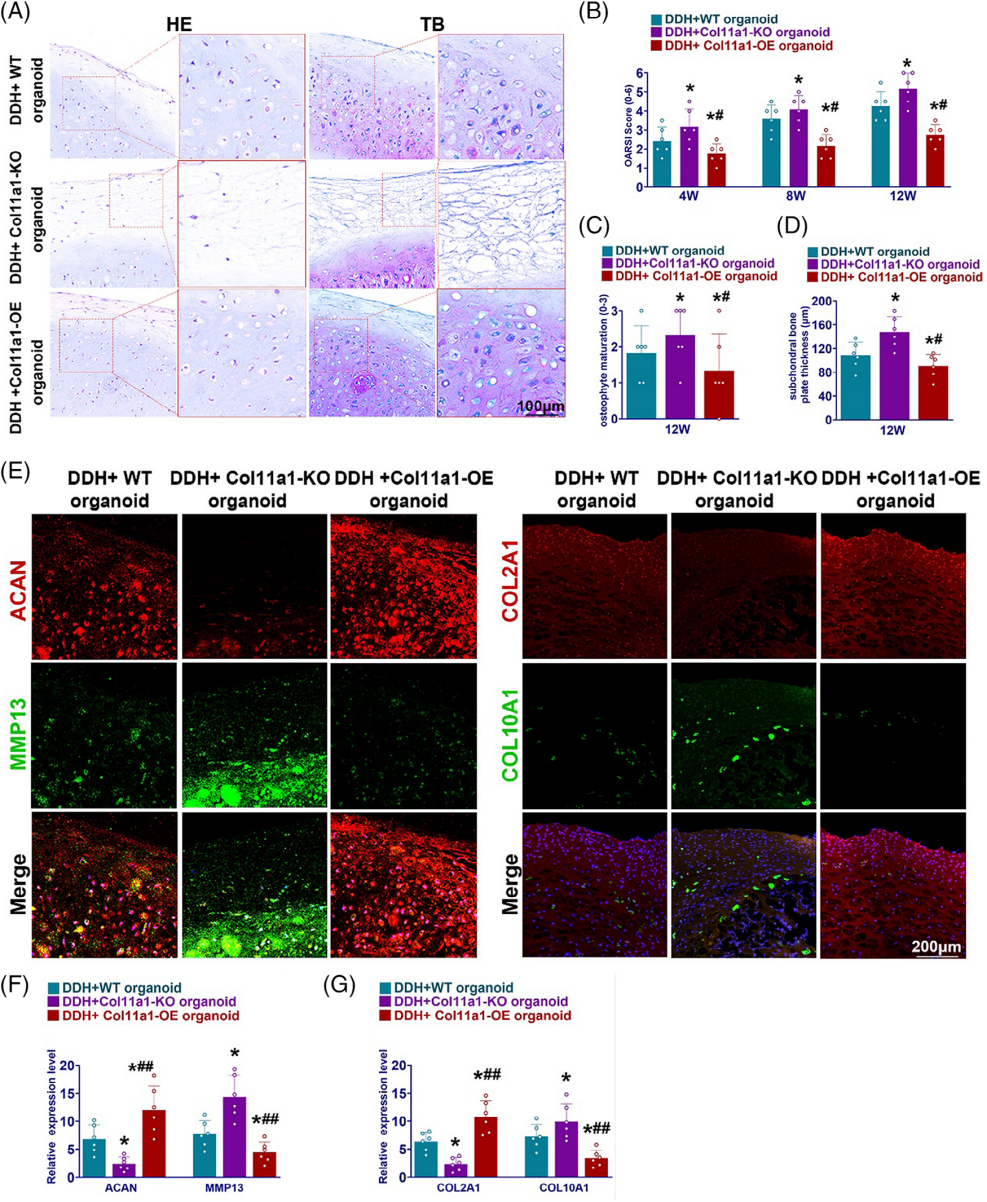
Local delivery of Col11a1‐OE organoids attenuated DDH‐induced joint degeneration in mice. (A) H&E and toluidine blue staining of femoral tissue in different groups. (B) Osteophyte maturation of femoral head in different groups at 12 weeks in DDH mice hip (*n* = 6 for each). (C) OARSI histopathological score of femoral head in different groups at 12 weeks in DDH mice hip (*n* = 6 for each). (D) Subchondral bone plate thickness at 12 weeks after in vivo injection in DDH mice hip (*n* = 6 for each). (E) Immunofluorescence staining of ACAN, MMP‐13, COL2A1 and COL10A1 expressions in femoral tissue at 12 weeks after in vivo injection in DDH mice hip (red, ACAN and COL2A1; green, MMP‐13 and COL10A1). (F and G) Relative ACAN, MMP‐13, COL2A1 and COL10A1 expression level in femoral head cartilage at 12 weeks after in vivo injection in DDH mice hip (*n* = 6 for each). *p < .05, **p < .01 compared with the DDH+WT organoid group, #*p* < .05, ##*p* < .01 compared with the DDH+Col11a1‐KO organoid group.

Col11a1‐OE organoids showed better chondrogenic properties and generated cartilaginous tissues in vitro. To demonstrate whether Col11a1‐OE organoids implantation in vivo could ameliorate the osteoarthritic progression of DDH, transplantation with intra‐articular injection of Col11a1‐KO organoids (DCKO group), Col11a1‐WT organoids (DCWO group) and Col11a1‐OE organoids (DCOO group) was performed for mice respectively with DDH induction surgery. After 12 weeks, we took samples of femoral head tissue to examine the joint protection abilities of different organoids. H&E staining showed Col11a1‐OE organoids maintain the cartilage tissue integrity and prevented cartilage degeneration in the femoral head (Figure 8A). Compared with DDH+Col11a1‐KO organoid group with severe cartilage damage, osteophyte forming and subchondral plate hyperplasia, injection of Col11a1‐OE organoids significantly protected joint cartilage as determined with histological assessments and better proteoglycan depositions (Figures 8B–D). Toluidine blue staining showed Col11a1‐OE organoids ameliorated fibrous tissue formation and cartilage degeneration in the femoral head (Figure 8A). The thickness of SBP was also significantly decreased in Col11a1‐OE group (Figure 8D). Immunofluorescence staining of joint cartilage showed greater ACAN and COL2A1 expression with much lower MMP13 and COL10A1 expression with injection of Col11a1‐OE organoids compared with Col11a1‐KO organoids (Figure 8E). Cartilage tissues of DDH+Col11a1‐OE organoid group also showed highest ACAN and COL2A1 expression with lowest MMP13 and COL10A1 expression levels in qRT‐PCR analysis (Figures 8F and G).

### 2.9 Col11a1‐OE organoids prevented DDH joint degeneration via regulating cellular senescence by up‐regulated Col11a1/HIF1α‐mediated glycolysis

**FIGURE 9:**
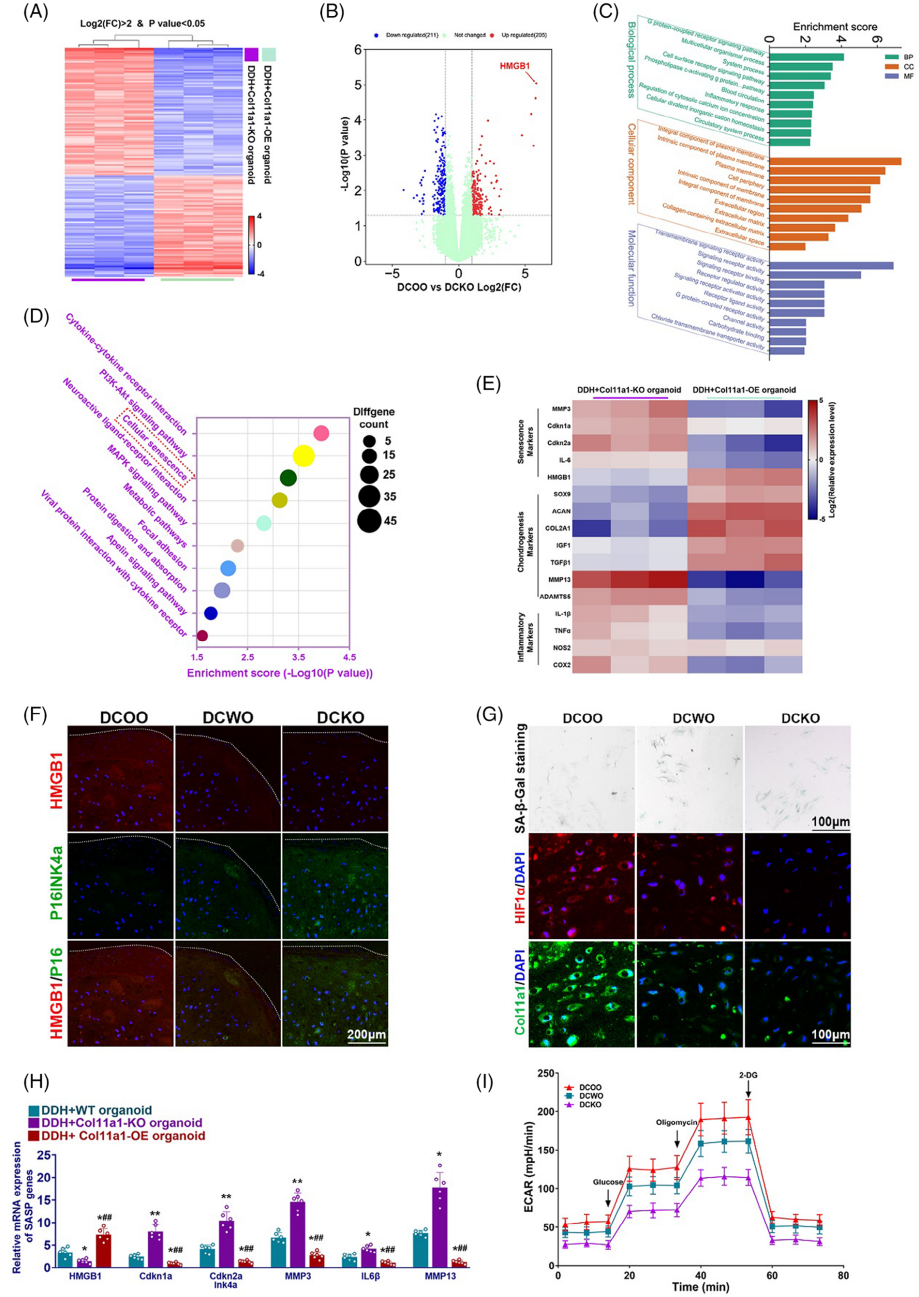
Col11a1‐OE organoids inhibits cellular senescence by up‐regulated cellular glycolysis to reverse DDH progression and joint degeneration in DDH mice. (A) Heatmap of clustering gene expression profiles with microarray in three DDH femoral head cartilage sample from DDH mice model with Col11a1‐OE organoid injected versus three DDH femoral head cartilage sample from DDH mice model with Col11a1‐KO organoid injected. (B) Volcano plot of gene expression profiles and HMGB1 was significantly up‐regulated in DDH mice model with Col11a1‐OE organoid injected. (C) Significantly enriched pathways for Col11a1‐OE organoids in Gene Ontology (GO) pathways. (D) Significantly enriched pathways for Col11a1‐OE organoids in Kyoto Encyclopedia of Genes and Genomes (KEGG) pathways. (E) Heatmap of clustering gene expression profiles with q‐PCR in three DDH femoral head cartilage sample from DDH mice model with Col11a1‐OE organoid injected versus three DDH femoral head cartilage sample from DDH mice model with Col11a1‐KO organoid injected. (F) Immunofluorescence staining of HMGB1 and P16INK4a in femoral head tissue in DDH mice injected with Col11a1‐OE, Col11a1‐KO and WT organoids. (G) SA‐β‐Gal staining (1st line) and immunostaining for HIF1α and Col11a1 (red, HIF1α; green, Col11a1; blue, DAPI) expression (2nd and 3rd lines) in chondrocytes derived from femoral head tissue in DDH mice injected with Col11a1‐OE, Col11a1‐KO and WT organoids. DDH mice injected with Col11a1‐KO organoids had highest level of SA‐β‐Gal staining. (H) Relative mRNA expression level of SASP gene in femoral head tissue in DDH mice DDH mice injected with Col11a1‐OE, Col11a1‐KO and WT organoids (*n* = 6 for each). (I) Extracellular acidification rate (ECAR) in glucose‐free medium was used to determine glycolytic metrics in collected chondrocytes in different groups, followed by sequential addition of glucose, oligomycin and 2‐DG (means ± SD, *n* = 6 for each). *p < .05, **p < .01 compared with the DDH+WT organoid group, #*p* < .05, ##*p* < .01 compared with the DDH+Col11a1‐KO organoid group.

To further investigate the regulatory mechanism and downstream of COL11A1 gene, we used gene sequencing and bioinformatic methods to clarify their complicated connection. Heatmap of clustering mRNA expression patterns with microarray in three DDH cartilage samples from Col11a1‐OE organoids injected mice versus three Col11a1‐KO organoids injected mice (Figure 9A). Volcano plot revealed HMGB1 up‐regulated significantly in DDH mice after Col11a1‐OE organoids treatment (Figure 9B). As far as we know, HMGB1 plays important roles in cellular senescence and inflammation. To demonstrate the exact role of cellular senescence in DDH development, GO pathways and KEGG pathways in tissue after Col11a1‐OE organoids injection were analysed (Figures 9C and D). Inflammatory response, regulation of cytosolic calcium ion concentration, extracellular region, extracellular matrix, collagen‐containing extracellular matrix, signalling receptor activity, signalling receptor binding and receptor regulator activity were significantly enriched in GO pathways (Figure 9C). Cellular senescence and protein digestion and absorption pathway were enriched in KEGG pathways (Figure 9D). To validate the senescent state of chondrocytes in the DDH cartilage samples, various senescence markers and inflammatory markers were analysed. RT‐PCR showed down‐regulation of MMP3, Cdkn2a, IL‐6, IL‐1β, TNF‐α, COX‐2 and up‐regulation of HMGB1, SOX9, ACAN, COL2A1, IGF1, TGF‐β1, indicating mitigation of state of senescence and inflammatory environment and facilitate chondrogenic circumstance (Figure 9E). Immunofluorescence staining showed that femoral head tissues after Col11a1‐OE organoids injection presented with abundant HMGB1 and significantly lower P16ink4a expression in DDH mice (Figures 9F and S5A and B). To further analyse the senescence state of the chondrocytes, SA‐β‐Gal staining was performed for collected chondrocytes in vitro, showing lower percent of SA‐β‐Gal positive chondrocytes after Col11a1‐OE organoids injection (Figures 9G and S5C). Immunostaining for HIF1α and Col11a1 expression indicated Col11a1‐OE organoids treatment significantly activated Col11a1–HIF1α signal in derived chondrocytes (Figure 9G). Dysregulation senescence markers (e.g., HMGB1, Cdkn1a, Cdkn2aink4a) and inflammation markers (e.g., MMP3, IL‐6b, MMP13) were further validated with qRT‐PCR, confirming treatment with Col11a1‐OE organoids remitted inflammation and prevented cellular senescent state of chondrocytes in DDH progression (Figure 9H). We then investigated key parameters of ECAR for the collected chondrocytes in different groups. Measured ECAR during the glycolysis stress test revealed the altered glycolytic patterns of chondrocytes with Col11a1‐KO or Col11a1‐OE organoid treatment compared with WT organoid treatment (Figure 9I). In addition, chondrocytes with Col11a1‐OE organoid treatment showed significantly increased glycolytic activities while DDH+Col11a1‐KO organoid samples exhibited significantly dampened glycolysis levels irrespective of the applied treatments (Figure 9I). These results further proved that Col11a1‐OE organoids could prevented DDH joint degeneration via regulating cellular senescence by up‐regulated glycolysis in vivo.

## 3 DISCUSSION

In this study, we reported that genome‐wide association between COL11A1 and DDH with large combined GWAS. Furthermore, WES of DDH pedigrees validated multiple mutations spanning the genomic regions of COL11A1. We further explored 3D‐cultured Col11a1‐OE SMSC organoids for joint degeneration treatment in DDH model. COL11A1 is one of the most significant genes influencing joint development. COL11A1 mutations were first identified in Marshall–Stickler syndromes.^17^, ^40^, ^41^ These mutation included c.1845+1G>A, c.2808+1G>C, c.3816+1G>A, c.3474+1G>A and c.1845 + 5G>C. A heterozygous deletion in COL11A1 involving exon 48, c.3627_3635del9 was identified in a skeletal dysplasia patient.^16^ Three mutations including c.2241+5G>T, c.2809–2A>G and c.3168+5G>C were identified to lead to type 2 Stickler syndrome and otospondylomegaepiphyseal dysplasia.^42^ However, COL11A1 mutation can not only result in joint dysplasia, but fibro‐chondrogenesis as well. Stuart W. Tompson et al.^43^ reported four mutations (c.2386G>C, c.3943G>T, c.1786dupG, c.3124G>A) in two fibrochondrogenesis patients. Several SNPs in COL11A1 gene have also been reported for joint degeneration. Meta‐analysis showed missense variation of COL11A1, SNP rs2126643 and rs3753841, were associated with osteoarthritis in Icelandic and UK population.^18^ Wang et al.^44^ reported rs1241164 and rs4907986 as hip OA risk loci in Chinese population, while rs1676486 were found associated with osteoarthritis development.^45^ Apart from single point mutation, Taylor et al.^46^ reported increased differentially hydroxymethylated regions of COL11A1 gene in osteoarthritic chondrocytes, indicating epigenetic regulation of COL11A1 expression in osteoarthritis. These previous reports indicated the indispensable role of COL11A1 in skeletal and joint development. Aggravated joint degeneration and OA phenotype was further observed in the present study for Col11a1‐defecient mice, confirming COL11A1 as a disease‐causing gene for DDH development. COL11A1 is an important gene affecting cartilage formation and joint morphogenesis. COL11A1 dysregulation was also found in incipient canine hip dysplasia.^20^ Hafez et al.^15^ found abnormal developing long bones and incompletely vertebral formation in the embryo of COL11A1 deficient mice. Meanwhile, COL11A1 plays key roles in fibril assembly, organisation and tendon function for joint morphogenesis.^14^ Abnormal fibril microstructure and fibrillar matrix deposition was identified in tendon of Col11a1‐knockout mice. These finding indicated COL11A1 might regulate bone and joint development by simultaneously regulating endochondral ossification and joint formation. In order to further demonstrate the concrete function of COL11A1 in our research, we set DDH+WT, DDH+COL11A1‐OE and DDH+COL11A1‐KO groups to analyse the impacts on the cartilage. The classical feature of DDH is the degeneration and then destruction of cartilage of femoral head because of mechanical abnormality it undertakes. In our results shown in Figures 7 and 8, despite the common DDH‐condition, SOX9 and ACAN were significantly down‐expressed in COL11A1‐KO model, which was contrast with WT and COL11A1‐OE groups. The result revealed the irreplaceable role of COL11A1 to protect the integrity of cartilage bearing abnormal stress induced by DDH. Together with the opposite trend of MMP13, the cytokine responsible of cartilage degradation, it could be asserted that COL11A1 plays a significant role in chondrogenic protection and maintaining the structure integrity of cartilage.

Using scRNA‐seq profiles for hip joint cartilage of DDH and Col11a1‐KO cartilage, we demonstrated disrupted chondrocyte homeostasis and cellular similarity in single‐cell transcriptomic analysis. Our results indicated that and Col11a1 deficiency could affect chondrocyte homeostatic state by dysregulated TCA cycle with declined glycolysis and increased OXPHOS, leading to aggravated cellular senescence and joint degeneration. Consistent with previous reports, our findings further provided evidence that glycolysis is the main metabolic energy source for chondrocytes and the shift in cellular energy metabolism towards increased OXPHOS contributed to cellular senescence with Col11a1 deficiency.^35^, ^36^, ^37^, ^38^, ^39^ Our scRNA‐seq results demonstrated that genetic deletion of Col11a1 significantly suppressed HIF1 signalling pathway. As an indispensable transcriptional regulator of chondrogenesis cellular glycolysis, HIF1α inactivation mediated glycolysis–OXPHOS shift in DDH and Col11a1‐KO chondrocytes.^24^ Col11a1/HIF1α axis activation appeared to be an anti‐senescent target for DDH treatment and OA prevention by regulating glycolysis–OXPHOS reprogramming in chondrocyte energy metabolism.

Our results demonstrated boosting Col11a1 expression might be of interest to potentiate cellular therapies for DDH and OA treatment by activating Col11a1–HIF1α–glycolysis axis in chondrocytes. Previous animal experiment also showed Col11a1‐OE mice demonstrated lower susceptibility to osteoarthritis and joint damage,^47^ indicating the therapeutic potential of COL11A1 in DDH treatment. Genetically inspired by the chondrogenic effects of COL11A1 in DDH development, we further generated Col11a1‐OE SMSC organoids to alleviate hip joint degeneration in DDH model. 3D‐cultrured organoids showed therapeutic potential in OA treatment in our previous study.^24^ Preclinical experiments of 3d‐cultured organoids showed better chondrogenesis in vitro and postponed OA degeneration in mice. However, cartilage and joint protection in DDH was much more challenging and no previous research so far have incorporated Col11a1‐OE SMSC in 3d‐cultured organoids for DDH treatment. COL11A1 overexpression better induced chondrogenesis of the SMSC organoids, and further suppress chondral hypertrophy and fibro‐chondral hyperplasia. Col11a1‐OE SMSC organoids offered a stable cellular microenvironment and protection of SMSC viability within the compacted organoids and promotes Col11a1‐induced chondrogenic differentiation. Lineage‐tracing studies have evidenced that COL11A1 is indispensable for the accurate formation and the cohesive properties of the cartilage collagen fibrils. COL11A1 deficiency might lead to overly fibro‐chondrogenesis caused by premature chondrocyte senescence, which is a well‐accepted risk factor in joint degeneration. In the present study, senescence markers were significantly down‐regulated in the cartilage tissues with Col11a1‐OE organoid treatment in the DDH model. Our results proved that transplanted Col11a1‐OE organoids in DDH could improve the inflammatory microenvironment and chondrocyte cellular senescence in the joint by activating the HIF1α–glycolysis axis. HMGB1, an antisenescence and anti‐inflammatory gene, has been discovered as a regulator of cellular proliferation, adhesiveness, and migration.^48^ HMGB1 was also identified and highly enriched in chondrocytes with Col11a1‐OE organoid treatment. We suppose HMGB1 might inhibited the infiltration of proinflammatory macrophage in joint degeneration, alleviated cellular stress to prevent cellular senescence and promoted chondrocyte homeostasis and proliferation. SA‐β‐Gal staining also confirmed the mitigatory status of senescent state after Col11a1‐OE organoid treatment, implicating that Col11a1‐OE organoid exerted its joint‐preserving effects by up‐regulating HIF1α/glycolysis‐mediated anti‐senescence. We have previously proved the effects of organoid injection for cartilage regeneration in our previously published study.(24) In this case, one limitation of our current study is that we did not include a ‘no organoid injection group’ when we want to focus on studying the effects of Col11a1 gene on organoid transplantation in the present study. Our study provided a new strategy with genetically engineered organoids targeting the Col11a1–HIF1α–glycolysis axis for DDH treatment and joint protection. For translation, we envision injectable Col11a1‐OE SMSC organoids in a surgery to incorporate DDH correction surgery to correct DDH deformation and prevent joint degeneration.

## 4 CONCLUSION

We reported genetic association between COL11A1 and DDH with combined GWAS and exome sequencing. Studies demonstrated involvement of COL11A1 expression was down‐regulated in DDH patients and Col11a1‐KO mice. ScRNA‐seq for DDH and Col11a1‐KO cartilage demonstrated disrupted chondrocyte homeostasis and cellular senescence caused by Col11a1–HIF1α‐mediated glycolysis–OXPHOS shift in chondrocytes. Genetically and biologically inspired, we further fabricated an intra‐articular injection therapy to preventing cartilage degeneration by generating a Col11a1‐OE SMSC organoids. Further experiments revealed that Col11a1‐OE organoids ameliorated cartilage degeneration in DDH mice via regulating cellular senescence by up‐regulated Col11a1/HIF1α‐mediated glycolysis in chondrocytes.

## AUTHOR CONTRIBUTIONS

R. H. and Q. W. contributed to conceiving the study and designing the experiments. Q. W. helped design the 3D‐bioprinted SMSC organoids and helped collect the sample of DDH patients and control. Y.‐Q. Y. helped perform the animal experiments. Y. S. and Y.‐Q. Y. analysed the data and wrote the manuscript. Y. S. and K.‐R. D. helped edit the manuscript and provided oversight. All authors read and approved the final manuscript.

## CONFLICT OF INTEREST STATEMENT

The authors have declared that no competing interest exists.

## FUNDING INFORMATION

The design of the study and collection, analysis and interpretation of data in this work was funded by the China National Natural Science Funds (No. 82172446).

## ETHICS STATEMENT

The ethical committee for Shanghai ninth people's hospital (school of medicine, Shanghai Jiao Tong university) approved all procedures (No. 2018053), and patients’ informed consent forms were obtained for publication.

## Supporting information

Supporting Information

## Data Availability

All data associated with this study are present in the paper or the supplementary materials.

## References

[1] Yang S , Zusman N , Lieberman E , Goldstein RY . Developmental dysplasia of the hip. Pediatrics. 2019;143(1):e20181147. 30587534 10.1542/peds.2018-1147

[2] Sewell MD , Rosendahl K , Eastwood DM . Developmental dysplasia of the hip. BMJ. 2009;339:b4454. 19934187 10.1136/bmj.b4454

[3] Woodacre T , Ball T , Cox P . Epidemiology of developmental dysplasia of the hip within the UK: refining the risk factors. J Child Orthop. 2016;10(6):633‐642. 27866316 10.1007/s11832-016-0798-5 PMC5145848

[4] Woodacre T , Dhadwal A , Ball T , Edwards C , Cox PJA . The costs of late detection of developmental dysplasia of the hip. J Child Orthop. 2014;8(4):325‐332. 24973899 10.1007/s11832-014-0599-7 PMC4128950

[5] Ortiz‐Neira CL , Paolucci EO , Donnon T . A meta‐analysis of common risk factors associated with the diagnosis of developmental dysplasia of the hip in newborns. Eur J Radiol. 2012;81(3):e344‐e351. 22119556 10.1016/j.ejrad.2011.11.003

[6] De Hundt M , Vlemmix F , Bais JMJ , et al. Risk factors for developmental dysplasia of the hip: a meta‐analysis. Eur J Obstet Gynecol Reprod Biol. 2012;165(1):8‐17. 22824571 10.1016/j.ejogrb.2012.06.030

[7] Panagiotopoulou N , Bitar K , Hart WJ . The association between mode of delivery and developmental dysplasia of the hip in breech infants: a systematic review of 9 cohort studies. Acta Orthop Belg. 2012;78(6):697‐702. 23409562

[8] Li L , Wang Xi , Zhao Q , et al. CX3CR1 polymorphisms associated with an increased risk of developmental dysplasia of the hip in human. J Orthop Res. 2017;35(2):377‐380. 27176135 10.1002/jor.23294

[9] Nejadhosseinian M , Haerian H , Shirkoohi R , Karami J , Mortazavi SMJ . Evaluation of CX3CR1 gene DNA methylation in developmental dysplasia of the hip (DDH). J Orthop Surg Res. 2022;17(1):436. 36175906 10.1186/s13018-022-03324-w PMC9523927

[10] Baghdadi T , Nejadhosseinian M , Shirkoohi R , et al. DNA hypermethylation of GDF5 in developmental dysplasia of the hip (DDH). Mol Genet Genomic Med. 2019;7(9):e887. 31338995 10.1002/mgg3.887 PMC6732267

[11] Hong L , Wang K , Yan W , et al. High performance immunochromatographic assay for simultaneous quantitative detection of multiplex cardiac markers based on magnetic nanobeads. Theranostics. 2018;8(22):6121‐6131. 30613287 10.7150/thno.29070 PMC6299706

[12] Shi D , Dai J , Ikegawa S , Jiang Q . Genetic study on developmental dysplasia of the hip. Eur J Clin Invest. 2012;42(10):1121‐1125. 22594447 10.1111/j.1365-2362.2012.02682.x

[13] Ma W , Zha Z , Chen K , et al. Genetic association study of common variants in TGFB1 and IL‐6 with developmental dysplasia of the hip in Han Chinese population. Sci Rep. 2017;7(1):10287. 28860542 10.1038/s41598-017-11185-1 PMC5579245

[14] Sun M , Luo EY , Adams SM , et al. Collagen XI regulates the acquisition of collagen fibril structure, organization and functional properties in tendon. Matrix Biol. 2020;94:77‐94. 32950601 10.1016/j.matbio.2020.09.001 PMC7722227

[15] Hafez A , Squires R , Pedracini A , Joshi A , Seegmiller R , Oxford J . Col11a1 regulates bone microarchitecture during embryonic development. J Dev Biol. 2015;3(4):158‐176. 26779434 10.3390/jdb3040158 PMC4711924

[16] Hufnagel SB , Weaver KN , Hufnagel RB , Bader PI , Schorry EK , Hopkin RJ . A novel dominant COL11A1 mutation resulting in a severe skeletal dysplasia. Am J Med Genet A. 2014;164A(10):2607‐2612. 25091507 10.1002/ajmg.a.36688 PMC9185704

[17] Guo L , Elcioglu NH , Wang Z , et al. Novel and recurrent COL11A1 and COL2A1 mutations in the Marshall‐Stickler syndrome spectrum. Hum Genome Var. 2017;4:17040. 28983407 10.1038/hgv.2017.40 PMC5628180

[18] Styrkarsdottir U , Lund SH , Thorleifsson G , et al. Meta‐analysis of Icelandic and UK data sets identifies missense variants in SMO, IL11, COL11A1 and 13 more new loci associated with osteoarthritis. Nat Genet. 2018;50(12):1681‐1687. 30374069 10.1038/s41588-018-0247-0

[19] Jiang H , Yang Q , Jiang J , Zhan X , Xiao Z . Association between COL11A1 (rs1337185) and ADAMTS5 (rs162509) gene polymorphisms and lumbar spine pathologies in Chinese Han population: an observational study. BMJ Open. 2017;7(5):e015644. 10.1136/bmjopen-2016-015644 PMC5623369 28583914

[20] Todhunter RJ , Garrison SJ , Jordan J , et al. Gene expression in hip soft tissues in incipient canine hip dysplasia and osteoarthritis. J Orthop Res. 2019;37(2):313‐324. 30450639 10.1002/jor.24178

[21] Sugimoto S , Kobayashi E , Fujii M , et al. An organoid‐based organ‐repurposing approach to treat short bowel syndrome. Nature. 2021;592(7852):99‐104. 33627870 10.1038/s41586-021-03247-2

[22] Sampaziotis F , Muraro D , Tysoe OC , et al. Cholangiocyte organoids can repair bile ducts after transplantation in the human liver. Science. 2021;371(6531):839‐846. 33602855 10.1126/science.aaz6964 PMC7610478

[23] Sun Ye , You Y , Jiang W , Zhai Z , Dai K . 3D‐bioprinting a genetically inspired cartilage scaffold with GDF5‐conjugated BMSC‐laden hydrogel and polymer for cartilage repair. Theranostics. 2019;9(23):6949‐6961. 31660079 10.7150/thno.38061 PMC6815949

[24] Sun Ye , Wu Q , Dai K , You Y , Jiang W . Generating 3D‐cultured organoids for pre‐clinical modeling and treatment of degenerative joint disease. Signal Transduct Target Ther. 2021;6(1):380. 34764239 10.1038/s41392-021-00675-4 PMC8585871

[25] Sun Ye , Wang C , Hao Z , et al. A common variant of ubiquinol‐cytochrome c reductase complex is associated with DDH. PLoS One. 2015;10(4):e0120212. 25848760 10.1371/journal.pone.0120212 PMC4388640

[26] Hatzikotoulas K , Roposch A , Wainwright A , et al. Genome‐wide association study of developmental dysplasia of the hip identifies an association with GDF5. Commun Biol. 2018;1:56. 30273415 10.1038/s42003-018-0052-4 PMC6123669

[27] Lek M , Karczewski KJ , Minikel EV , et al. Analysis of protein‐coding genetic variation in 60,706 humans. Nature. 2016;536(7616):285‐291. 27535533 10.1038/nature19057 PMC5018207

[28] Booth KT , Askew JW , Talebizadeh Z , et al. Splice‐altering variant in COL11A1 as a cause of nonsyndromic hearing loss DFNA37. Genet Med. 2018;21(4):948‐954. 10.1038/s41436-018-0285-0 PMC6431578 30245514

[29] Matsuzaki T , Alvarez‐Garcia O , Mokuda S , et al. FoxO transcription factors modulate autophagy and proteoglycan 4 in cartilage homeostasis and osteoarthritis. Sci Transl Med. 2018;10(428):eaan0746. 29444976 10.1126/scitranslmed.aan0746 PMC6204214

[30] Kreuser U , Buchert J , Haase A , Richter W , Diederichs S . Initial WNT/beta‐catenin activation enhanced mesoderm commitment, extracellular matrix expression, cell aggregation and cartilage tissue yield from induced pluripotent stem cells. Front Cell Dev Biol. 2020;8:581331. 33195222 10.3389/fcell.2020.581331 PMC7661475

[31] Kennedy D , Mnich K , Oommen D , et al. HSPB1 facilitates ERK‐mediated phosphorylation and degradation of BIM to attenuate endoplasmic reticulum stress‐induced apoptosis. Cell Death Dis. 2017;8(8):e3026. 29048431 10.1038/cddis.2017.408 PMC5596589

[32] Wei T , Kulkarni NH , Zeng QQ , et al. Analysis of early changes in the articular cartilage transcriptisome in the rat meniscal tear model of osteoarthritis: pathway comparisons with the rat anterior cruciate transection model and with human osteoarthritic cartilage. Osteoarthritis Cartilage. 2010;18(7):992‐1000. 20434574 10.1016/j.joca.2010.04.012

[33] Ji Q , Zheng Y , Zhang G , et al. Single‐cell RNA‐seq analysis reveals the progression of human osteoarthritis. Ann Rheum Dis. 2019;78(1):100‐110. 30026257 10.1136/annrheumdis-2017-212863 PMC6317448

[34] Chen Y , Yu Y , Wen Ya , et al. A high‐resolution route map reveals distinct stages of chondrocyte dedifferentiation for cartilage regeneration. Bone Res. 2022;10(1):38. 35477573 10.1038/s41413-022-00209-w PMC9046296

[35] Mobasheri A , Rayman MP , Gualillo O , Sellam J , Van Der Kraan P , Fearon U . The role of metabolism in the pathogenesis of osteoarthritis. Nat Rev Rheumatol. 2017;13(5):302‐311. 28381830 10.1038/nrrheum.2017.50

[36] Arra M , Swarnkar G , Ke Ke , et al. LDHA‐mediated ROS generation in chondrocytes is a potential therapeutic target for osteoarthritis. Nat Commun. 2020;11(1):3427. 32647171 10.1038/s41467-020-17242-0 PMC7347613

[37] Matsuoka K , Bakiri L , Bilban M , et al. Metabolic rewiring controlled by c‐Fos governs cartilage integrity in osteoarthritis. Ann Rheum Dis. 2023;82(9):1227‐1239. 37344157 10.1136/ard-2023-224002 PMC10423482

[38] Wu X , Fan X , Crawford R , Xiao Y , Prasadam I . The metabolic landscape in osteoarthritis. Aging Dis. 2022;13(4):1166‐1182. 35855332 10.14336/AD.2021.1228 PMC9286923

[39] Nishida T , Kubota S , Aoyama E , Takigawa M . Impaired glycolytic metabolism causes chondrocyte hypertrophy‐like changes via promotion of phospho‐Smad1/5/8 translocation into nucleus. Osteoarthritis Cartilage. 2013;21(5):700‐709. 23384547 10.1016/j.joca.2013.01.013

[40] Mladenova M , Todorov T , Grozdanova L , Mitev V , Todorova A . Novel mutation in the COL11A1 gene causing Marshall‐Stickler syndrome in three generations of a Bulgarian family. Balkan J Med Genet. 2021;24(1):95‐98. 10.2478/bjmg-2021-0001 PMC8366474 34447665

[41] Brizola E , Gnoli M , Tremosini M , et al. Variable clinical expression of Stickler Syndrome: a case report of a novel COL11A1 mutation. Mol Genet Genomic Med. 2020;8(9):e1353. 32558342 10.1002/mgg3.1353 PMC7507508

[42] Micale L , Morlino S , Schirizzi A , et al. Exon‐trapping assay improves clinical interpretation of COL11A1 and COL11A2 intronic variants in Stickler syndrome type 2 and otospondylomegaepiphyseal dysplasia. Genes (Basel). 2020;11(12):1513. 33348901 10.3390/genes11121513 PMC7766184

[43] Tompson SW , Bacino CA , Safina NP , et al. Fibrochondrogenesis results from mutations in the COL11A1 type XI collagen gene. Am J Hum Genet. 2010;87(5):708‐712. 21035103 10.1016/j.ajhg.2010.10.009 PMC2978944

[44] Wang J , Zhang C , Wu SG , et al. Additional evidence supports association of common variants in COL11A1 with increased risk of hip osteoarthritis susceptibility. Genet Test Mol Biomarkers. 2017;21(2):86‐91. 27936936 10.1089/gtmb.2016.0308

[45] Raine EVA , Dodd AW , Reynard LN , Loughlin J . Allelic expression analysis of the osteoarthritis susceptibility gene COL11A1 in human joint tissues. BMC Musculoskelet Disord. 2013;14:85. 23497244 10.1186/1471-2474-14-85 PMC3599795

[46] Taylor SEB , Li YH , Wong WH , Bhutani N . Genome‐wide mapping of DNA hydroxymethylation in osteoarthritic chondrocytes. Arthritis Rheumatol. 2015;67(8):2129‐2140. 25940674 10.1002/art.39179 PMC4519426

[47] Holyoak DT , Otero M , Armar NS , et al. Collagen XI mutation lowers susceptibility to load‐induced cartilage damage in mice. J Orthop Res. 2018;36(2):711‐720. 28898438 10.1002/jor.23731 PMC8813548

[48] Riuzzi F , Sorci G , Donato R . The amphoterin (HMGB1)/receptor for advanced glycation end products (RAGE) pair modulates myoblast proliferation, apoptosis, adhesiveness, migration, and invasiveness. Functional inactivation of RAGE in L6 myoblasts results in tumor formation *in vivo* . J Biol Chem. 2006;281(12):8242‐8253. 16407300 10.1074/jbc.M509436200

