# Supplementary material for: Genetically inspired organoids prevent joint degeneration and alleviate chondrocyte senescence via Col11a1–HIF1α‐mediated glycolysis–OXPHOS metabolism shift
Source: Clin Transl Med. 2024 Feb 5;14(2):e1574. doi: 10.1002/ctm2.1574 (PMC10840017; doi:10.1002/ctm2.1574)
Supplement: Supplementary file 1 — Supporting Information [file CTM2-14-e1574-s001.docx]

**Supplementary Materials**

**Methods and Materials**

**GWAS of DDH patient samples**

Separate GWAS studies of hip dysplasia in the NJR and Chinese population have been conducted, including 1156 hip dysplasia patients and 3922 controls.[1, 2] Genotype data of the promising loci in Col11A1(rs3753841 and rs145901197) was extracted from the GWAS studies. A further replication study for the two loci above with 218 DDH cases and 360 controls was conducted in Chinese Han population. A meta-analysis combining the replication study and the GWAS data (1374 cases, 4282 controls) was conducted to explore the association between the DDH and Col11A1 loci within SAS software (version 9.2 - SAS Institute, Cary, NC, USA). We used the Mantel-Haenszel method and a random-effects model according to the method of DerSimonian and Laird.[3] We evaluated heterogeneity using I^2^ statistics,[4] interpreting a value of I2 less than 50% as substantial heterogeneity. When I^2^ statistics indicated substantial heterogeneity using a fixed effects model, we considered intra-cluster homogeneity as not assessed. We thus used a random-effects model. Bilateral chi square tests were conducted to determine the significance of differences in allelic frequencies and P < 0.05 was considered to be statistically significant.

**Whole exome sequencing for familiar DDH pedigrees**

To further explore rare variants of Col11A1 gene in hip dysplasia heritability, exome sequencing for familiar DDH patients was conducted in different populations (17 patients in 8 Chinese families, 9 patients in 3 Saudi Arabia families) using Agilent SureSelect Whole Exome capture and Illumina sequencing technology. A variant was considered to be a candidate mutation if it was de novo mutation or present in affected family patients, was absent in the controls, and had not previously been reported or if it had a prevalence below 0.1% in the 1000 Genomes variant database and the Exome Aggregation Consortium. Candidate variants in Col11A1 were extracted from the exome sequencing data of the 11 DDH families. Crosscheck of the mutation frequency in gnomAD[5] was made for identified variants. Finally, for the remaining candidate mutation sites, the potential influence of the mutations on protein functions was predicted using sorting intolerant from tolerant (SIFT©) algorithm and Mutation-Taster© to help determine the most possibly responsible mutations for DDH.

DDH patients were consecutively recruited from the department of orthopedics of Ninth Hospital, which is affiliated with Shanghai Jiao Tong University. Controls were recruited from physical examination center in the Ninth Hospital. Joint samples of cartilage and synovial tissues were obtained from 6 male control (averaging 49.5 years old; KL score=0, 3 patients; KL score=1, 3 patients) patients with leg amputation and 21 male DDH patients (averaging 53.7 years old; KL score=1, 3 patients; KL socre=2, 6 patients; KL score=3, 6 patients; KL socre=4, 6 patients) undergoing hip arthroplasty. Joint degeneration severity was calculated with OARSI histopathological scores. The study protocol was approved by the Ethics Committee of Shanghai ninth people’s hospital and the first affiliated hospital of Nanjing medical university. All methods were performed in accordance with the relevant guidelines and regulations. Informed consents were obtained from all participants of the study. The study was approved by the Ninth Hospital and the medical school of Shanghai Jiao Tong University Ethics Committee and informed consents were obtained from all patients and controls. All methods were performed in accordance with the relevant guidelines and regulations

**Genotyping of targeted locus**

According to the manufacture’s protocol, the DNA of all the subjects was extracted either from the buccal swabs using the DNA IQ System (Promega, Madison, WI) or peripheral blood using the NucleoSpin Blood QuickPure Kit (Macherey-Nagel GmbH & Co. KG, Düren, German). All the samples were genotyped with Taqman assay. The sample was genotyped by uninformed laboratory personnel. Genotyping, data input and statistical results were examined by two authors independently. Five percent samples were randomly selected to repeat, and 100% consistency was obtained.

**Cell Expansion and Formation of organoids**

Mouse SMSCs and chondrocytes were isolated from synovial biopsies and joint cartilage of healthy mouse hip joints. Mouse chondrocytes were isolated from joint cartilage. Briefly, Joint cartilage was harvested and immediately transferred into plastic tubes. Isolated chondrocytes were expanded in 37 °C, 5% O_2_, and 95% humidity in α-MEM containing 10% FBS, 4.5 mg/mL D-glucose,0.1 mM nonessential amino acids, 1 mM sodium pyruvate, 100 mM Hepes buffer, 100 Ul/mL penicillin, 100 μg/mL streptomycin, and 0.29 mg/mL L-glutamate. Medium was changed twice a week and primary chondrocytes were used for the following experiments . The SMSC pools were expanded until passage 3 for organoid formation at 37 °C, 5% CO_2_, and 95% humidity in Dulbecco’s modified Eagle medium (DMEM, Life Technologies, China) with 10% fetal bovine serum (HyClone FBS, Thermo Scientific, USA), 1% antibiotic– antimycotic (100 units mL^−1^ penicillin, 100 mg mL^−1^ streptomycin, and 0.25 mg mL^−1^ amphotericin B), and 1 × 10^−3^ M sodium pyruvate (Life Technologies, China). Medium was changed every 2–3 days, and SMSCs were harvested with Trypsin (Life Technologies, China) at a confluence of 80–90%. Trypsin was used for all passaging and harvesting steps during cell handling. The ethical committee for Shanghai ninth people’s hospital (school of medicine, Shanghai Jiao Tong university) approved all procedures, and patients’ informed consent forms were obtained.

Agarose microwell inserts for formation of a high number of SMSC organoids with homogeneous size distribution were created as previously described by Leijten et al[6]. Briefly, 3 % (w/v) Agarose (Invitrogen, China) was poured onto a polydimethylsiloxane (PDMS, Corning Sylgard 184 elastomer, MAVOM Chemical Solutions) master mould containing 200 µm-diameter pillars. The agarose was let to solidify where after microwell inserts with an area of ≈1.8 cm^2^ were punched out, placed in 24-well plates, 1 mL of phosphate-buffered saline (PBS) was added, and the wells were sterilized under UV for 30 min. Each well insert contained nearly 2000 microwells. SMSCs were harvested and seeded with a concentration of 1000 000 cells per well to obtain ≈500 cells per organoid after self-aggregation. SMSC organoids were differentiated into cartilaginous microtissues in a serum-free chemically defined chondrogenic medium (CM) containing LG-DMEM (Gibco) supplemented with 1% antibiotic–antimycotic (100 units mL^−1^ penicillin, 100 mg mL^−1^ streptomycin, and 0.25 mg mL^−1^ amphotericin B), 1 × 10^−3^ M ascorbate-2 phosphate, 100 × 10^−9^ M dexamethasone, 40 µg mL^−1^ proline, ITS+ Premix Universal Culture Supplement (Corning) (including 6.25 µg mL^−1^ insulin, 6.25 µg mL^−1^ transferrin, 6.25 µg mL^−1^ selenious acid, 1.25 µg mL^−1^ bovine serum albumin (BSA), and 5.35 µg mL^−1^ linoleic acid), 100 ng mL^−1^ growth/ differentiation factor 5 (GDF5) (PeproTech) and 10 ng mL^−1^ TGF-β3 (PeproTech)[7], Half of the media volume was changed every 2-3 days. Cell viability in SMSC organoids was assessed qualitatively with LIVE/DEAD Viability/Cytotoxicity Kit (Invitrogen) by following the manufacturer’s protocol as previously reported. Cell nucleus and F-actin distribution within SMSC organoids were visualized by staining with 2.5 µg mL^−1^ 4’,6-diamidino-2-phenylindole (DAPI) (Invitrogen) and 0.8 U mL^−1^ Alexa Fluor 488 phalloidin (Invitrogen) during 1 h at room temperature. Stained organoids were imaged with an inverted laser scanning fluorescence confocal microscope with 1 µm thick slices as previously reported[6].

**Microarray analysis**

Microarray analysis of 6 cartilage samples (3 DDH+COL11A1-OE organoids vs 3 DDH+COL11A1-KO organoids) was performed using an Agilent mRNA Microarray Kit, Release 21.0, 8x60K (Agilent Technologies, CA, USA). Total RNA was quantified by a NanoDrop ND-2000 (Thermo Scientific, SA, USA), and RNA integrity was assessed using an Agilent Bioanalyzer 2100 (Agilent Technologies). Sample labeling, microarray hybridization, and washing were performed according to the manufacturer’s protocols. Briefly, total RNA was dephosphorylated, denatured, and then labeled with cyanine-3-CTP. After purification, the labeled RNAs were hybridized into the microarray. After being washed, the arrays were scanned with an Agilent Scanner G2505C (Agilent Technologies). Feature Extraction software (version 10.7.1.1, Agilent Technologies) was used to analyze the array images to obtain raw data. Next, Genespring software (version 14.8, Agilent Technologies) was utilized to finish the basic analysis of the raw data. First, the raw data were normalized with the quantile algorithm. The probes that had at least 100.0 percent of samples in any 1 condition out of 2 conditions with flags in "Detected" were chosen for further data analysis. Differentially expressed mRNAs were then identified using R software (Version 3.6.1) with the “limma” package through fold change (FC) and adjusted P value. The threshold for up- and down-regulated genes was set at an FC > 4.0 and an adjusted P value < 0.05. The differentially expressed mRNAs were further validated by quantitative reverse transcriptase–polymerase chain reaction (qRT-PCR). Gene ontology (GO) analysis and Kyoto Encyclopedia of Genes and Genomes (KEGG) analysis were applied to determine the roles of these target genes by R. Hierarchical clustering were performed to show the differential mRNA expression patterns among samples using scatter plots, volcano plots, and heatmaps for visualization.

**Animal experiments**

**Ectopic cartilage formation in vivo**

The animal experiment protocols were approved by Shanghai Ninth People’s Hospital, medical school of Shanghai Jiao Tong University Ethics Committee and the local Institutional Animal Care and Use Committee (IACUC) and complied with the Guide for the Care and Use of Laboratory Animals published by the National Academy Press (National Institutes of Health Publication No. 85-23, revised 1996). SMSCs or induced SMSC organoids were suspended in α-MEM containing 10% FBS. Then, 100 μl of the cell suspension was injected subcutaneously into the dorsal flank of 6-week-old female nude mice. Mice were sacrificed after 4 weeks, and the injected sites were dissected from the mice. The samples were fixed in 4% paraformaldehyde, processed, and embedded in paraffin. Serial sections (4-μm thick) of the generated ectopic cartilage were cut through the center of the injection site and stained with toluidine blue and safranin-O according to standard protocols. Immunohistochemical staining of chondrocyte markers ACAN and COL2A1 were conducted according to standard protocols in the generated cartilage tissue sections in different groups. The stained images were taken using a light microscope. GAGs and types XI collagen were quantitatively assayed (6 vs 6) and normalized to DNA content. GAG production and COL XI expression were compared among different treatment groups.

**Injection of SMSC mini-organoids for OA treatment in mice hip joint**

Mice were used to examine the effect of SMSC mini-organoids for experimental DDH in vivo. Femoral head dislocation was performed in 4-week-old mice to construct a DDH model. X ray of the dislocated hip joint was taken 12 weeks after DDH induction in mice. Four-week-old wild-type (WT) (Col11a1^+/+^) and homozygous cho (Col11a1^−/−^) on a C57Bl6 background were used to conduct DDH model and further analyzed. Mice were randomized into seven groups (n=6 for each group; two hips of each mouse were used): wide-type mice with DDH surgery, wide-type mice without DDH surgery, COL11A1-KO mice without surgery, COL11A1-KO mice with DDH surgery, DDH+WT organoid group with wide type SMSC 3D-cultured organoids injection, DDH + COL11A1-OE organoid group with COL11A1 overexpression SMSC 3D-cultured organoids injection, DDH + COL11A1-KO organoid group with COL11A1 knock out SMSC 3D-cultured organoids injection. After the operation, mice were allowed to move freely in their single cages and fed with standard food and water. Femoral head of the hip joint was collected at different time points. Serial sections (4-μm thick) were cut sagittally through the center of the most diseased DDH site and stained with H&E and Safranin-O & SA-β-Gal according to standard protocols. Immunohistochemical staining of cartilage markers (ACAN and MMP13) and senescence markers (HMGB1 and P16INK4a) were also conducted according to standard protocols in the generated cartilage tissue sections in different groups compared to the native cartilage. The stained images were taken using a light microscope. Histological assessment of sagittal sections of the knee joints was conducted by two blinded observers who followed the Osteoarthritis Research Society International (OARSI) scoring system[8]. Measurements were also performed for osteophyte maturation, synovitis score (0-3, 0 = no synovial thickening; 1 = lining of two cell layers; 2 = several extra cell layers; 3= clear inflammation with cell infiltrate or exudate) and subchondral bone plate thickness (the region between the osteochondral junction and marrow space on the medial side of the tibial plateau) using Bioquant Osteo software (BIOQUANT, Inc.) as describe previously[9, 10].

**Immunofluorescence staining of histological sections**

To evaluate the distribution and expression of proteins, immunofluorescence analysis was performed as described by the previous reports[11]. In brief, frozen sections of mice colons with a thickness of 6μm were fixed in 1% paraformaldehyde and washed using PBS. After blocking with 5% normal goat serum diluted in PBS, sections were then incubated with primary antibodies in PBS with 1% goat serum (4℃, overnight). After washing with PBS, sections were incubated with secondary antibodies for 1 h. Image visualization was performed using confocal microscopy (Olympus, Tokyo, Japan). The primary antibodies in this study were available upon reasonable request (Table S1).

**RNA isolation, cDNA synthesis, and qRT-PCR**

Total RNA isolation from joint tissues or SMSC cells was performed using Trizol reagent (Invitrogen, Carlsbad, CA, USA) according to the manufacturer’s instructions. RNA quality and quantity determination were performed using a bioanalyzer (Agilent Inc., Santa Clara, CA, USA) and nanodrop (Thermo Scientific). For quantitative detection of miRNA and mRNA, RT-PCR was performed using a qSYBR-green-containing PCR kit (Qiagen, Germantown, MD, USA) with an RT-PCR system (Applied Biosystems, SA, USA). U6 small nuclear RNA (snRNA) and GAPDH were used as controls for normalization. mRNA qRT-PCR primers and internal control were purchased from Applied Biosystems. All PCR assays were performed in triplicate using the 2^-△△Ct^ method. All Primer sequences are available from the authors upon request.

**Single-cell RNA-seq and data processing**

After the acquirement, fresh cartilage tissues from different groups were washed by sterile PBS for three times. The hip joint cartilage tissues were minced to 1–2 mm pieces and digested with 0.2% collagenase II (#2195526, Gibco, USA) diluted in DMEM solution at 37 °C for 4 h. Then, the cell suspension was filtered with 70 μm strainer to remove the incompletely digested cartilage ECM. After centrifuged, chondrocytes were suspended in DPS buffer (#14190144, Thermo Fisher, USA) and their viability was assessed by Countess® II Automated Cell Counter (Thermo Fisher).The Cell Ranger software pipeline (version 5.0.0) provided by 10×Genomics was used to demultiplex cellular barcodes, map reads to the genome and transcriptome using the STAR aligner, and down-sample reads as required to generate normalized aggregate data across samples, producing a matrix of gene counts versus cells. We processed the unique molecular identifier (UMI) count matrix using the R package Seurat[12] (version 4.0.0). To remove low-quality cells and likely multiplet captures, which is a major concern in microdroplet-based experiments, a set of criteria were conducted: Cells were filtered by (1) gene numbers (gene numbers < 200), (2) UMI (UMI <1000), (3) log10GenesPerUMI (log10GenesPerUMI < 0.7), (4) percentage of mitochondrial RNA UMIs (proportion of UMIs mapped to mitochondrial genes > 10%) and (5) percentage of hemoglobin RNA UMIs (proportion of UMIs mapped to hemoglobin genes > 5%). Subsequently, we applied DoubletFinder package[13] (version 2.0.2) to identify potential doublet. After applying these QC criteria, 19514 single cells were included in downstream analyses. To obtain the normalized count, library size normalization was processed using NormalizeData function in Seurat[12]. Specifically, the global-scaling normalization method “LogNormalize” normalized the gene expression measurements for each cell by the total expression, multiplied by a scaling factor (10,000 by default), and the results were log-transformed.

Top variable genes across single cells were identified using the method described in Macosko et al[14], were selected using FindVariableGenes function(mean.function = FastExpMean, dispersion.function = FastLogVMR) in Seurat[12]. Principal-component analysis (PCA) was performed to reduce the dimensionality with RunPCA function in Seurat[12]. Graph-based clustering was performed to cluster cells according to their gene expression profile using the FindClusters function in Seurat[12]. Cells were visualized using a 2-dimensional Uniform Manifold Approximation and Projection (UMAP) algorithm with the RunUMAP function in Seurat[12]. We used the FindAllMarkers function (test.use = presto) in Seurat[12] to identify marker genes of each cluster. FindAllMarkers identified positive markers for a given cluster compared with all other cells. Then, we used the R package SingleR[15] (version 1.4.1), a novel computational method for unbiased cell type recognition of scRNA-seq, with the reference transcriptomic datasets ‘SCMCA’ to infer the originating cell of each single cell independently and identify their cell types. Differentially expressed genes(DEGs) were selected using the FindMarkers function (test.use = presto) in Seurat[12]. P value < 0.05 and |log2foldchange| > 0.58 was set as the threshold for significantly differential expression.

GO enrichment and KEGG pathway enrichment analysis of DEGs were respectively performed using R based on the hypergeometric distribution. The sequencing and bioinformatics analysis were provided by BOAO Biotech Co., Ltd. (Shanghai, China).

**Glycolysis stress test**

The glycolysis stress test and the mito stress test were performed on murine primary chondrocytes with Seahorse Bioscience XF Analyzer (Agilent Tech) following the instructions of manufacturer. Briefly, the murine chondrocytes were seeded in the XF96 cell culture microplate (Seahorse Bioscience, 101085-004) with 100,000 cells per well. Ahead of the assays, the culture medium was replaced followed by 1h incubation in 37℃. For the glycolysis stress test, cell culture medium was replaced by Seahorse XF Base medium (Seahorse Bioscience), supplemented with L-glutamine. During the assay, glucose, oligomycin and 2-deoxyglucose were added into each well sequentially, followed by mixing and measurements. Mixture time, incubation time and the timeline of chemicals addition were determined based according to instructions of manufacturer. The glycolytic function of the cells was determined. Calculations were made using the average of all measurements per injection and normalized to total protein content using BCA protein assay (Pierce, Thermo Fisher Scientific). Data were analyzed using Wave Desktop 2.6 (Agilent Technologies).

**In vitro siRNA transfection**

Mouse SMSCs and chondrocytes were transfected with COL11A1 overexpression, knockout plasmid, or negative control using Lipofectamine 3000 (Invitrogen) according to the manufacturer's instructions. The COL11A1 expression plasmid was obtained using the pcDNA™3.1/V5-His TOPO™ TA Expression Kit (Invitrogen™). After 48 h of transfection, the expression levels of the target genes were evaluated from the collected cellular lysates by qRT-PCR and western blotting.

**Beta-galactosidase staining**

SA–β-gal staining was done using a SA–β-gal staining kit (catalog no. 9860; Cell Signaling Technology, Danvers, MA, USA) according to the manufacturer's instructions. First, culture medium was removed from the cells.and the plate was rinsed with 1X PBS (2 ml or a 35 mm well plate, or match volume of media) . Fixative Solution(1X, 1 ml) was added to each 35 mm well to allow cells to fix for 10-20 min at room temperature. Then the plate was rinsed again two times with 1X PBS. After that, 1 ml of the β-Galactosidase Staining Solution was added to each 35 mm well for incubation at 37°C overnight in a dry incubator (without CO2). Cells were checked for the development of blue color under a microscope (200X total magnification). Images were taken under the microscope, and β-Galactosidase staining solution was removed and the cells were overlaid with 70% glycerol for long- term storage of the plates at 4°C.

**Cell immunofluorescence**

Cell immunofluorescence analysis was performed according to the descriptions by the previous studies[16]. Briefly, SMSCs or induced organoids were fixed in 4% PFA, permeabilized with PBS containing 0.5% Triton X-100 for 20 min, and then blocked with 3% BSA containing 0.025% Triton X-100 and 5% FBS at room temperature for 30 min. Cells were immune-stained via incubation with primary antibodies (Table S1) at 4°C overnight. After that, the cells were washed using PBS three times and incubated with appropriate secondary antibodies. After the cells were washed, DAPI was used for nuclear counterstaining for 5 min. Immunofluorescence visualization was performed using a confocal microscope (Carl Zeiss, Germany). Each experiment was conducted in triplicate, and representative confocal microscopy images are shown.

**Statistical analysis**

The software of SPSS (Version 19.0, SPSS Inc., Chicago, IL, USA) and GraphPad Prism (Version 8.0, GraphPad Software Inc., San Diego, CA, USA) were used for statistical analysis. Mann–Whitney U test, Student’s t-test, and one-way ANOVA test were used for data analysis as appropriate. Pearson’s correlation analysis was performed for the correlation between relative miR-138 expression and OA progression in clinical osteoarthritic patients. P < 0.05 was considered statistically significant.

1. Sun Y, Wang C, Hao Z, Dai J, Chen D, Xu Z, et al. A common variant of ubiquinol-cytochrome c reductase complex is associated with DDH. PLoS One. 2015; 10: e0120212.

2. Hatzikotoulas K, Roposch A, Consortium DDHCC, Shah KM, Clark MJ, Bratherton S, et al. Genome-wide association study of developmental dysplasia of the hip identifies an association with GDF5. Commun Biol. 2018; 1: 56.

3. DerSimonian R, Laird N. Meta-analysis in clinical trials. Control Clin Trials. 1986; 7: 177-88.

4. Higgins JP, Thompson SG, Deeks JJ, Altman DG. Measuring inconsistency in meta-analyses. BMJ. 2003; 327: 557-60.

5. Lek M, Karczewski KJ, Minikel EV, Samocha KE, Banks E, Fennell T, et al. Analysis of protein-coding genetic variation in 60,706 humans. Nature. 2016; 536: 285-91.

6. Leijten J, Teixeira LS, Bolander J, Ji W, Vanspauwen B, Lammertyn J, et al. Bioinspired seeding of biomaterials using three dimensional microtissues induces chondrogenic stem cell differentiation and cartilage formation under growth factor free conditions. Sci Rep. 2016; 6: 36011.

7. Mendes LF, Tam WL, Chai YC, Geris L, Luyten FP, Roberts SJ. Combinatorial Analysis of Growth Factors Reveals the Contribution of Bone Morphogenetic Proteins to Chondrogenic Differentiation of Human Periosteal Cells. Tissue Eng Part C Methods. 2016; 22: 473-86.

8. Gerwin N, Bendele AM, Glasson S, Carlson CS. The OARSI histopathology initiative - recommendations for histological assessments of osteoarthritis in the rat. Osteoarthritis Cartilage. 2010; 18 Suppl 3: S24-34.

9. Zhen G, Wen C, Jia X, Li Y, Crane JL, Mears SC, et al. Inhibition of TGF-beta signaling in mesenchymal stem cells of subchondral bone attenuates osteoarthritis. Nat Med. 2013; 19: 704-12.

10. Kim JH, Jeon J, Shin M, Won Y, Lee M, Kwak JS, et al. Regulation of the catabolic cascade in osteoarthritis by the zinc-ZIP8-MTF1 axis. Cell. 2014; 156: 730-43.

11. Clayburgh DR, Barrett TA, Tang Y, Meddings JB, Van Eldik LJ, Watterson DM, et al. Epithelial myosin light chain kinase-dependent barrier dysfunction mediates T cell activation-induced diarrhea in vivo. J Clin Invest. 2005; 115: 2702-15.

12. Butler A, Hoffman P, Smibert P, Papalexi E, Satija R. Integrating single-cell transcriptomic data across different conditions, technologies, and species. Nat Biotechnol. 2018; 36: 411-20.

13. McGinnis CS, Murrow LM, Gartner ZJ. DoubletFinder: Doublet Detection in Single-Cell RNA Sequencing Data Using Artificial Nearest Neighbors. Cell Syst. 2019; 8: 329-37 e4.

14. Macosko EZ, Basu A, Satija R, Nemesh J, Shekhar K, Goldman M, et al. Highly Parallel Genome-wide Expression Profiling of Individual Cells Using Nanoliter Droplets. Cell. 2015; 161: 1202-14.

15. Aran D, Looney AP, Liu L, Wu E, Fong V, Hsu A, et al. Reference-based analysis of lung single-cell sequencing reveals a transitional profibrotic macrophage. Nat Immunol. 2019; 20: 163-72.

16. Nguyen HT, Dalmasso G, Muller S, Carriere J, Seibold F, Darfeuille-Michaud A. Crohn's disease-associated adherent invasive Escherichia coli modulate levels of microRNAs in intestinal epithelial cells to reduce autophagy. Gastroenterology. 2014; 146: 508-19.

**Table S1. Details of the antibodies used in the present study**

| **Antibodies** | **manufacturer** | **Product code** | **Concentration used** |
| --- | --- | --- | --- |
| Aggrecan（human，mouse） | SAB | 45068-1 | 1:200 |
| COL11A1（human，mouse） | SAB | 40770-1 | 1:200 |
| GLUT1 | proteintech | 21829-1-AP | 1:800 |
| COX17 | proteintech | 11464-1-AP | 1:500 |
| HMGB1 | Affinity Biosciences | AF7020 | 1:500 |
| P16INK4A | abcam | ab211542 | 1:200 |
| HIF1α | abcam | ab308433 | 1:100 |


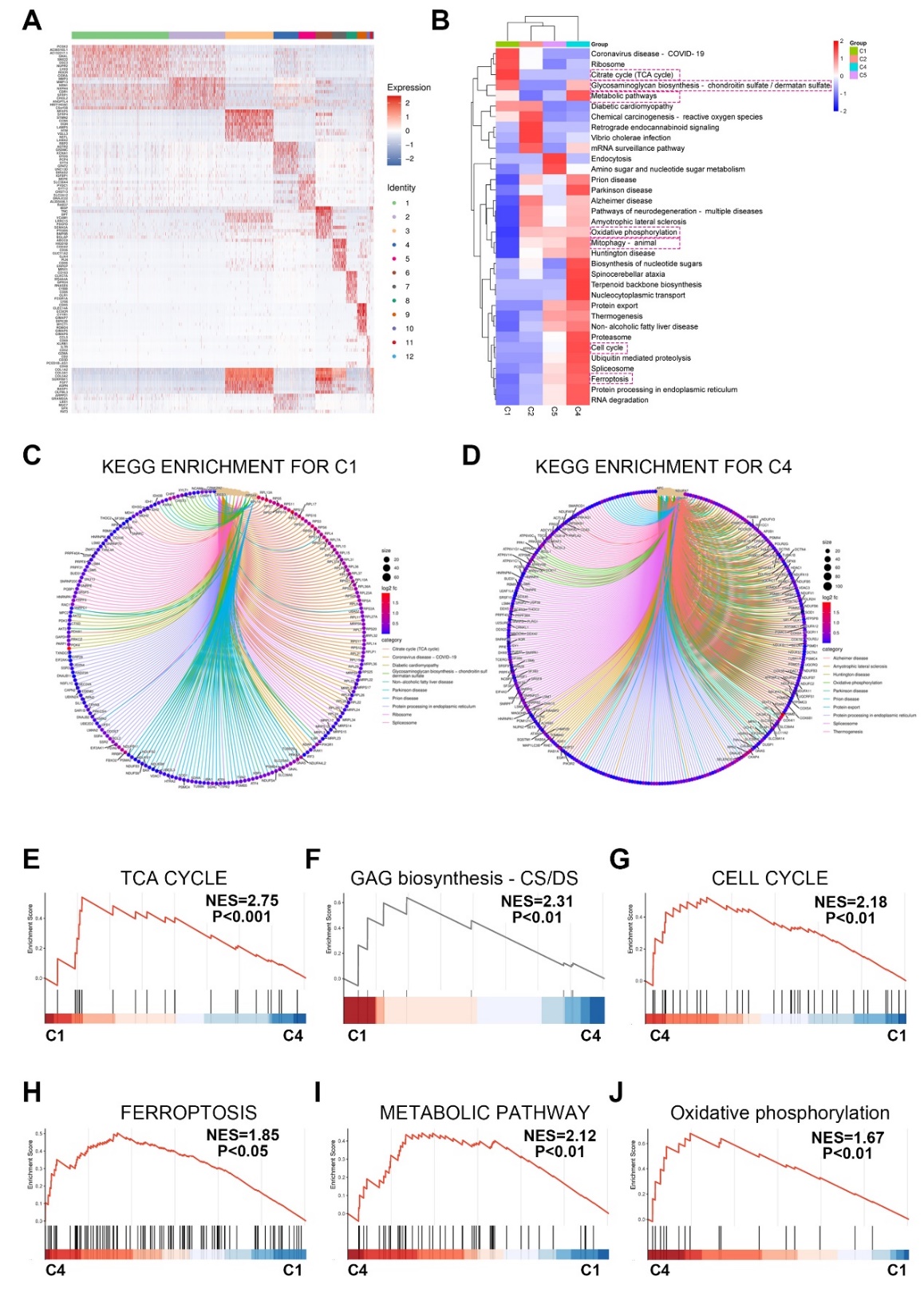


**Figure S1.** **A.** Heatmap revealing the scaled expression of preferentially and differentially expressed genes for each cluster. **B.** The heatmap of Kyoto Encyclopedia of Genes and Genomes (KEGG) analysis showing differentially enriched signaling pathways among the four main chondrocyte clusters. The representative pathway terms were marked with frames of red dotted line. **C-D.** Cnetplot revealed the pathway-gene connection for representative KEGG pathways in C1 and C4**. E-J.** Gene set enrichment analysis (GSEA) revealing the enrichment of representative function terms in C1 and C4 clusters. NES, normalized enrichment score; P, P value.

**\**

**
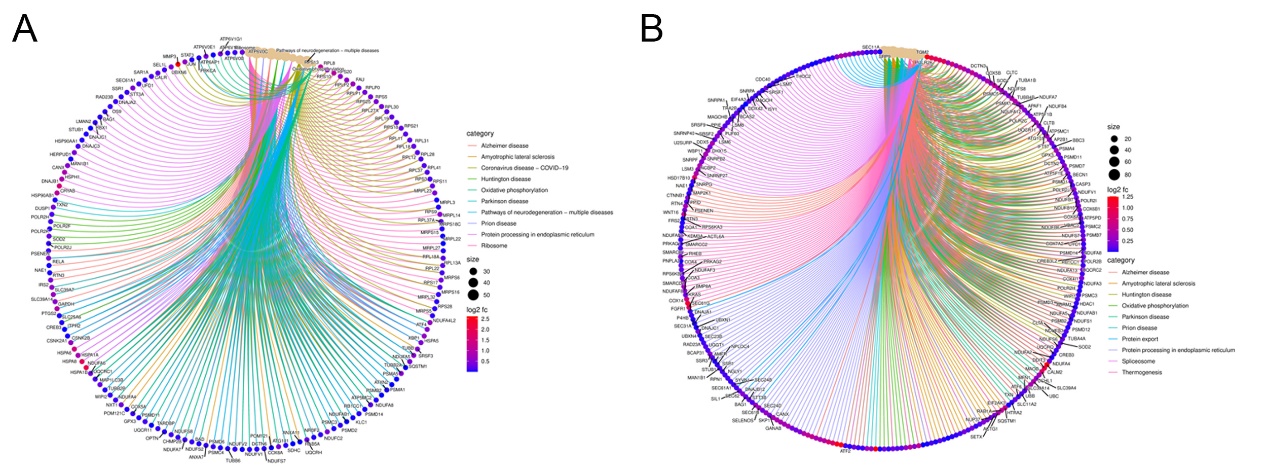
**

**Figure S2. A.** Cnetplot revealed the pathway-gene connection for representative KEGG pathways in C2 **B.** Cnetplot revealed the pathway-gene connection for representative KEGG pathways in C5


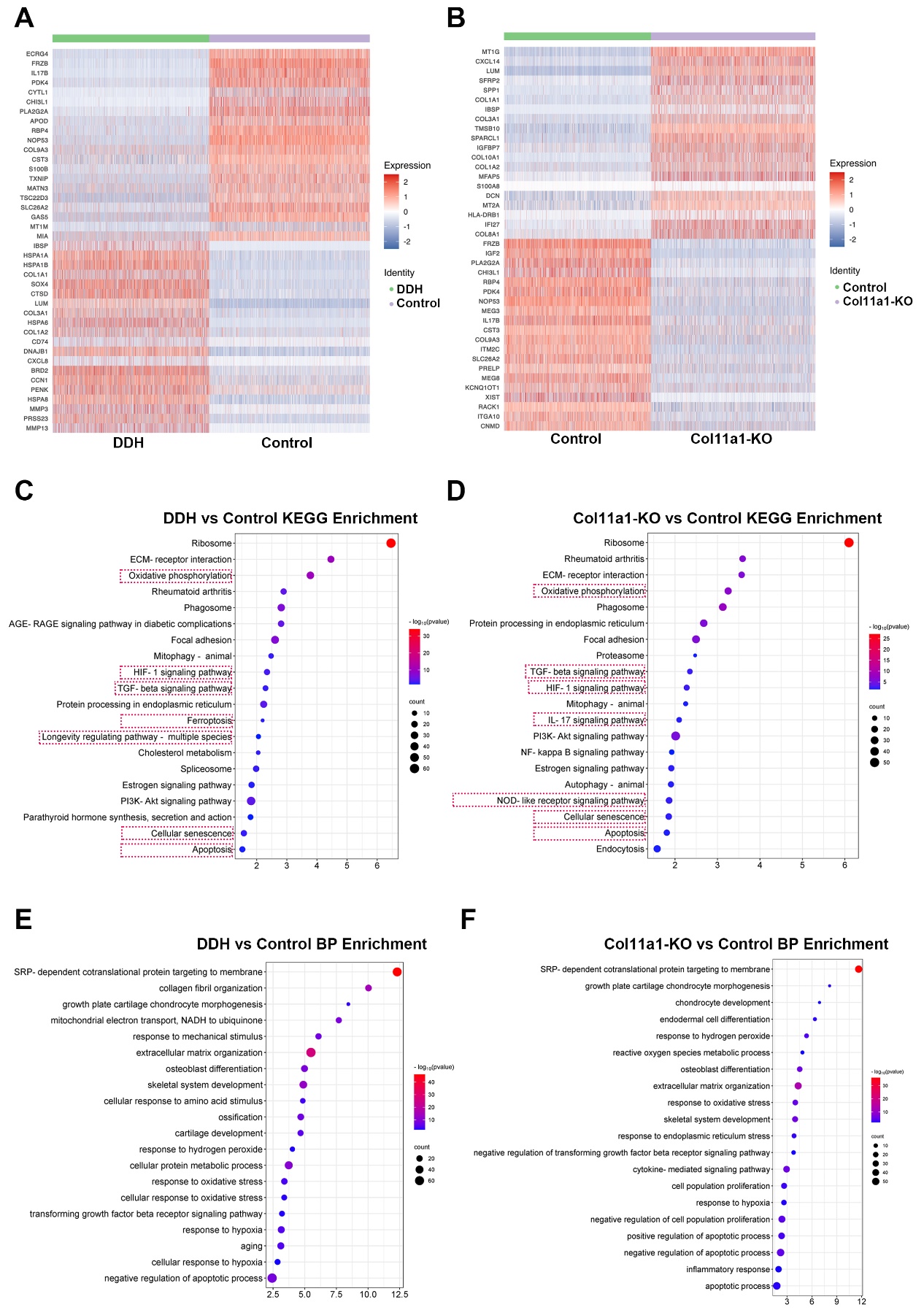


**Figure S3. A-B.** Heatmap revealing the scaled expression of preferentially and differentially expressed genes for DDH and Col11a1 cartilage compared to control. **C-D.** Heatmap of KEGG analysis showing differentially enriched signaling pathways in DDH and Col11a1-KO cartilage compared to control. The representative pathway terms were marked with frames of red dotted line. **E-F.** Enrichment of Gene Ontology (GO) analysis revealed representative biological process (BP) terms in DDH and Col11a1-KO cartilage compared to control**.**

**
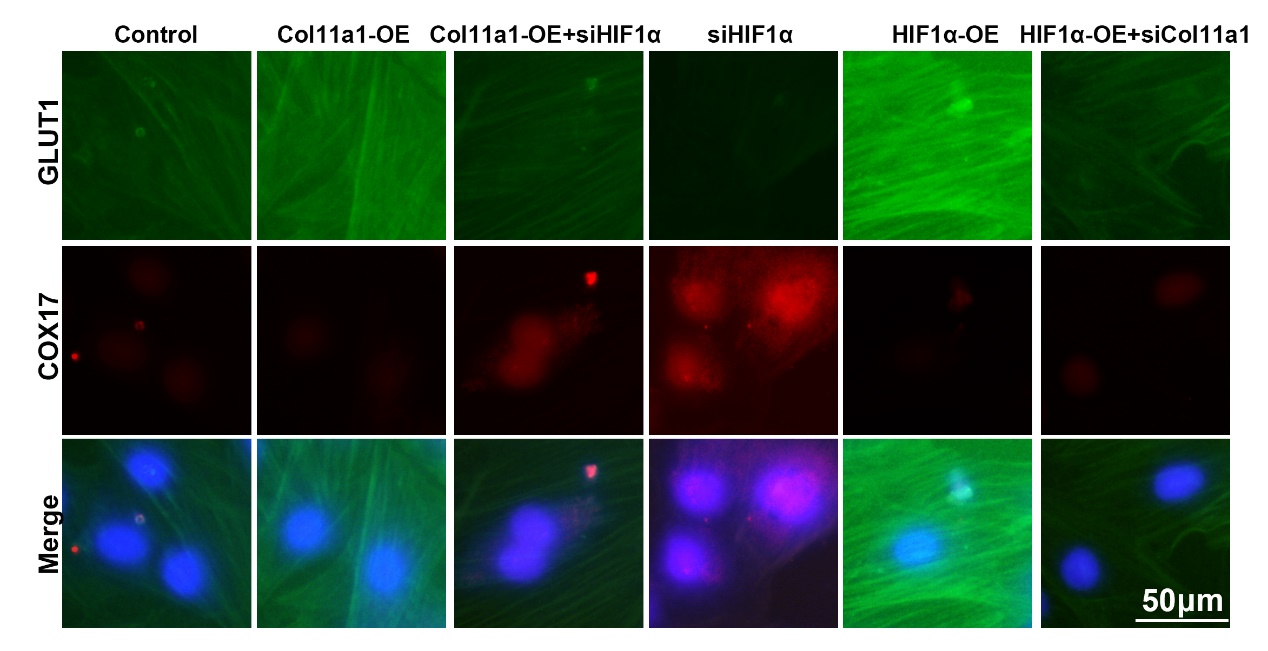
**

**Figure S4.** Immunofluorescence staining of HMGB1 and P16 in cultured chondrocytes transfected with HIF1α, Col11a1 small interfering RNA (siHIF1α and siCol11a1) construct, Lenti-Col11a1 (Col11a1-OE) and Lenti-HIF1α (HIF1α-OE) respectively in vitro. Col11a1-OE and HIF1α-OE chondrocytes were further transfected with siHIF1α and siCol11a1 respectively to verify the Col11a1/HIF1α axis for HMGB1 and P16 expression.


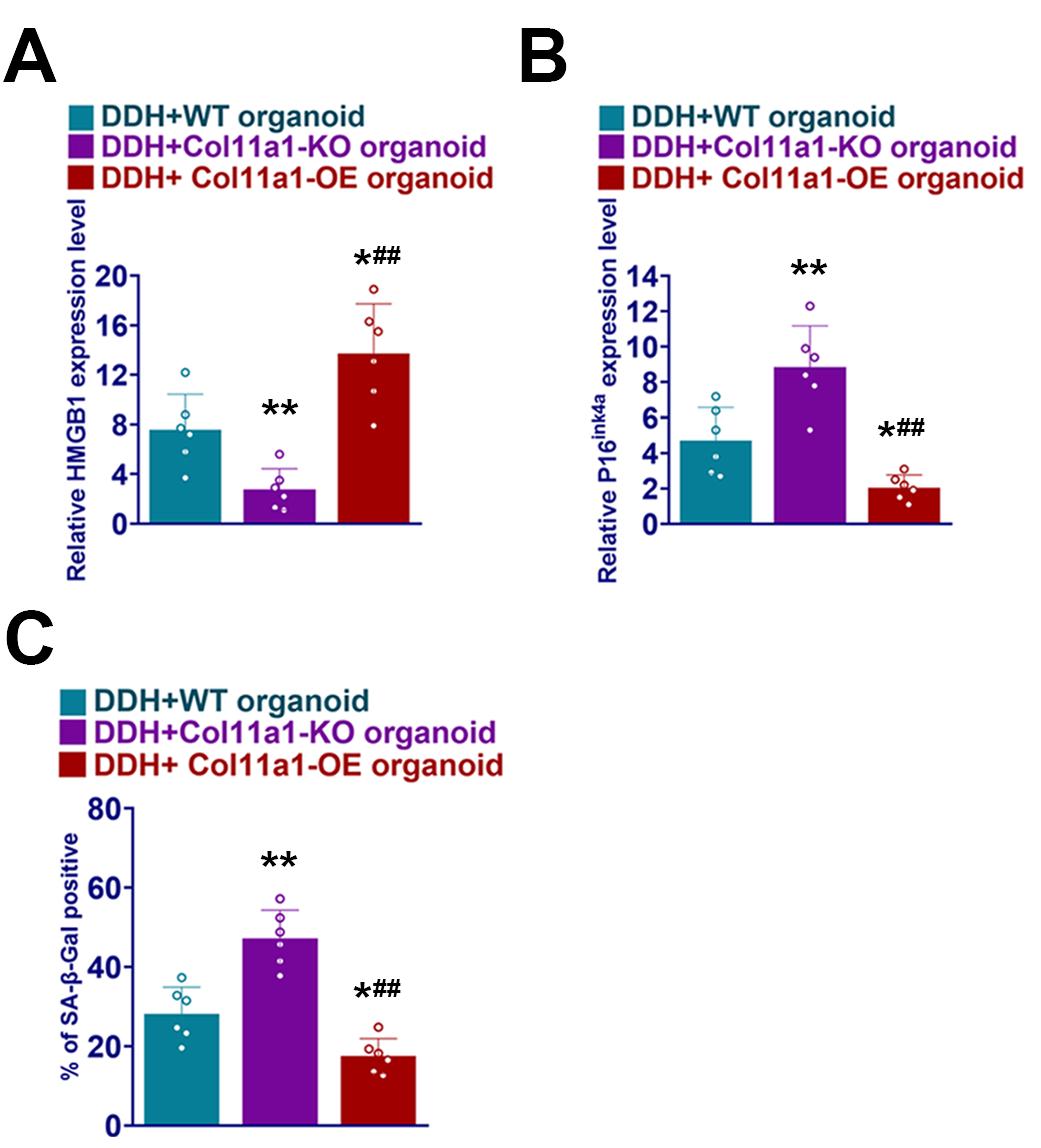
**Figure S5.** **A-B.** Relative HMGB1 and P16INK4a expression level in femoral head tissue in DDH mice DDH mice injected with Col11a1-OE, Col11a1-KO and WT organoids. (n=6 for each) **C.** Percent of SA-β-Gal positive cells in femoral head tissue in DDH mice DDH mice injected with Col11a1-OE, Col11a1-KO and WT organoids. (n=6 for each) *P < 0.05, **P < 0.01 compared to the DDH+WT organoid group, #p < 0.05, ##p<0.01 compared to the DDH+Col11a1-KO organoid group.
